# Stimuli-Responsive Carriers for Delivery of Anticancer Bioactive Agents

**DOI:** 10.3390/ma19071400

**Published:** 2026-03-31

**Authors:** Mariusz Gadzinowski, Stanislaw Slomkowski, Teresa Basinska

**Affiliations:** Division of Functional Polymers and Polymer Materials, Centre of Molecular and Macromolecular Studies, Polish Academy of Sciences, H. Sienkiewicza 112, 90-363 Lodz, Poland; mariusz.gadzinowski@cbmm.lodz.pl (M.G.); stanislaw.slomkowski@cbmm.lodz.pl (S.S.)

**Keywords:** stimuli-responsive, delivery carrier, biodegradable polymer, nanoparticles, anticancer drug, amphiphilic copolymer

## Abstract

The review describes advances in stimulus-sensitive carriers for chemotherapy of various organs, since selectivity in cytotoxicity against cancer and normal cells is a key factor in effective cancer treatment. Special attention is devoted to particle carriers composed of natural compounds, such as lipids, phospholipids, oligopeptides, and synthetic macromolecules, that are sensitive to internal or external stimuli, and delivered to targeted body tissue in a controlled manner. The stimuli discussed include the following: temperature, pH, enzymes, electromagnetic radiation, ultrasound, and redox potential. The description of stimulus-sensitive drug delivery, the methods for synthesizing polymers and copolymers, and the preparation of nano- and microparticles are briefly presented. A description of drug delivery systems (DDSs) with controlled release to specific organs, such as the breast, intestine, lung, prostate, etc., is preceded by a description of methods for preparing drug carriers. The review also covers DDSs at various stages of preclinical and clinical trials and summarizes the state of knowledge on this subject.

## 1. Introduction

According to the World Health Organization (WHO), “In 2022, there were an estimated 20 million new cancer cases and 9.7 million deaths. The estimated number of people who were alive within 5 years following a cancer diagnosis was 53.5 million. About 1 in 5 people develop cancer in their lifetime, and approximately 1 in 9 men and 1 in 12 women die from the disease. The data cited above indicate the need to intensify research in developing new, better therapies for cancer treatment.

The vast majority of anticancer bioactive substances are cytotoxic to both cancer and normal cells. Consequently, their use as drugs requires formulations with very high cytotoxicity against cancer cells and extremely low cytotoxicity against normal cells for preparation. High selectivity can be achieved thanks to carriers that ensure that encapsulated bioactive compounds are isolated from interactions with normal cells during prolonged circulation in body fluids, most often in the blood, and during their targeted delivery to tumors or other cancer cell systems. Once at the target site, the carriers should be effectively captured by the cancer cells and would release their cytotoxic cargo. In some cases, a carrier should also contain bioactive compounds that reduce the efflux of the cytotoxic compound from the cancer cells. If properly selected, stimuli control the processes mentioned above and facilitate more efficient progress. For this purpose, suitable stimulus-sensitive carriers should be used. Of course, the material for these carriers, as for many others, should be non-toxic, non-immunogenic, non-inflammatory, and non-irritating. Ideally, it should degrade after the required time to avoid accumulation during long enough administration. Functionalized metal nanoparticles (NPs), mesoporous silica, liposomes (LPs), and various constructs made from synthetic and natural polymers are used as drug carriers. Polymers were chosen as drug carriers because they can be obtained in many ways. In addition, polymers may have a wide range of tailored properties. The carrier’s surface can be equipped with ligands that bind to receptors on tumor cells’ surfaces, such as folic acid, transferrin, and biotin. The ligands improve selective tumor-targeted binding, facilitating the direct delivery of anti-cancer drugs.

The review focuses on stimulus-sensitive polymer carriers for chemotherapy, since selectivity in cytotoxicity toward cancer and normal cells is crucial in this field.

It should be emphasized, however, that although there are reviews in the literature on stimulus-responsive systems, this work presents a unique and different perspective on the issue.

The article discusses advances in preparing different classes of stimuli-sensitive carriers and compares them, considering their advantages and disadvantages. Furthermore, it describes DDSs applied to treatment in various types of cancer in specific organs.

It also discusses recent studies on stimulus-sensitive drug formulations at the proof-of-concept level, in vitro cell culture studies, and in vivo preclinical studies in animal models. The following sections describe the procedures fulfilled to introduce a new drug formulation to the market and the review of formulations in various phases of clinical trials. Finally, perspectives on stimulus-sensitive anticancer drug formulations are presented.

## 2. Basic Remarks on Stimulus-Sensitive Systems

The release of drugs by particles is strictly controlled. After being delivered to the body, the particles (anticancer DDS) are usually transported by the blood to their destination; in rarer cases, they are applied directly to the tumor.

DDSs have been developed that act (release active compounds) at the site of the cancer (usually a tumor) under the influence of external (outside the body) and internal (inside the body) stimuli. External factors include various electromagnetic waves and ultrasound. Internal factors within the body that utilize the local and specific properties of tumor tissue in the design of DDSs include pH, enzymes, and receptors on the surface of tumor cells.

The factors remain unchanged at the target site (tumor), while the factors that enable drug release act actively or passively. Passive action involves the spontaneous release of the drug at the target site, which is influenced by the surrounding tumor tissue environment.

The stimuli-responsive systems respond to internal stimuli present in cancer cells, such as glutathione (GSH), enzymes, redox potential, reactive oxygen species (ROS), pH, and external stimuli (working from external sources, in the vicinity of the cancer in the body), such as temperature, electromagnetic field, and ultrasound.

**Figure 1:**
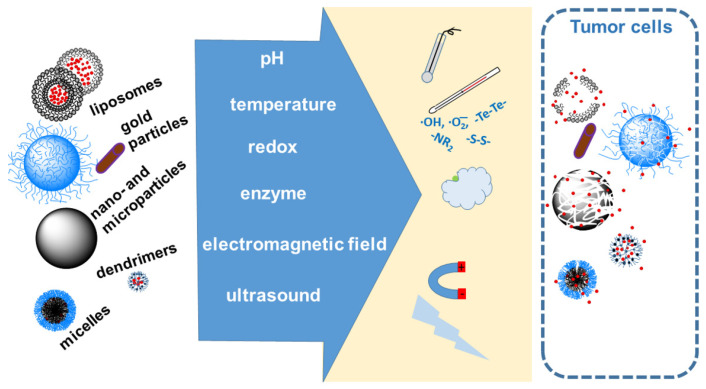
Overview of stimuli-sensitive systems described in the paper.

The most commonly used stimuli controlling the behavior of anticancer drug carriers, described in this paper, are shown in Figure 1.

Although the chemical structure (or structures) of drug carriers in all the mentioned systems can be similar, the individual segments of the compounds (usually polymer chains) differ and are prone to change in response to a given stimulus. Mostly, the stimulus is active “on wish” and affects the collapse, decay, or hydrolysis of the prone chemical bound within the polymer chain. Stimulus-responsive DDSs release drugs in a controlled, continuous, or dose-controlled manner, with concentrations remaining within the therapeutic range for an extended time or rapidly upon activation. However, it is worth stressing that due to the complexity of the cancer, a general recipe to fight it does not exist.

The nano- or microparticles used in stimuli-sensitive systems are usually composed of a hydrophobic core and a hydrophilic shell. The core of the particle serves as a warehouse for the anti-cancer drug. The hydrophilic coating, on the other hand, comes into contact with the environment and should prevent rapid elimination from the body and non-specific interactions, such as inflammation. The copolymers synthesized for responsive stimuli systems are complex and usually composed of di-, tri-, or multi-blocks. The anti-cancer drug can be physically adsorbed within the particles’ interior. However, the precise control of drug release requires the active compound to be covalently bound to the copolymer matrix. The drug is stimulus-induced by the particle in the target tumor tissue.

It is worth noting that many types of carriers with anticancer drugs do not reach the central part of the tumor but accumulate in the vicinity of the tumor’s blood vessels. To overcome this barrier, the size, shape, and surface morphology of the particles should be optimized, and measures should be developed to improve the quality of the tumor’s blood vessels and modify the tumor’s extracellular matrix to facilitate drug transport within the tumor [1]. Particle shrinking may be a solution for deeper tumor penetration in one case or charge or shape reversal in another. The comprehensive review on this topic describes many nanoparticle DDSs and shows various relationships between particle properties and their actions and functions in tumor tissue [1]. The modification can concern not only the properties of the carrier itself but also the embedded drug molecule [2,3].

An important point is also the ligand tethered to the carrier’s surface that binds specifically to receptors on cancer cells. The most commonly applied ligands in drug delivery carriers are folic acid, targeted peptides, and proteins, like low density lipoprotein, transferrin, cell-penetrating cyclic nonapeptides, monoclonal antibodies, etc. [1].

The particles used in stimulus-sensitive systems are composed of lipids, phospholipids, polymers, and copolymers that contain hydrophilic and hydrophobic segments or that form various hierarchical structures, such as nanogels (NGs) [4] or dendrimers (DDRs) [5,6,7].

From a chemical point of view, polymers used in the manufacture of DDSs, especially hydrophobic particle cores, belong to polyesters (polycarbonates [8], polylactides [9], poly(ε-caprolactone), polysiloxanes), polyamides (polypeptides [10]), or mixtures/segments of the abovementioned compounds. The particle shell usually contains poly(ethylene glycol) (PEG) (i.e., poly(ethylene oxide)), with a molecular weight not exceeding 20 kDa), and linear or cyclic polysaccharides. The polymers/copolymers used in the systems are both natural and synthetic. The natural polymers are oligo- or polysaccharides, like dextrans, hyaluronan, alginate derivative [11], lipids [12], phospholipids, e.g., lysophosphatidylcholine [13], and combinations of lipids with oligosaccharides, forming glycolipids, peptides belonging to various classes, like collagen, and elastin [14,15].

pH and temperature are the stimuli that influence the hydrophobicity of polymers in drug carriers stabilized by intermolecular interactions [16].

The pH of healthy tissue is 7.4 and decreases to 6.5 in the tumor microenvironment, i.e., cancer cells’ organelles, such as nucleosomes, decrease to 5.5 and to 5.0 in lysosomes [17].

In a pH-sensitive DDSs, the drug can be triggered by the protonation of chemical groups along polymer chains caused by the lower pH in diseased cancer tissue. The commonly used ones are tertiary amine and sulfonamide. Another approach is the introduction acid-labile chemical linkages into the polymer chain, such as imines, hydrazones, orthoesters, or acetals, which remain stable at pH = 7.4 but break apart in acidic conditions in cancer tissue. As the pH decreases, the drug carrier disintegrates or undergoes chain rearrangement, releasing bioactive molecules. The pH is decreased in the cancer microenvironment due to lactic acid overproduction during glycolysis, whereas oxidative phosphorylation occurs in healthy tissue [18].

Temperature also plays an important role as a stimulus-reactive factor, especially for polymers exhibiting a lower critical solubility temperature (LCST). For polymers with an LCST below the critical temperature, the polymer is miscible with water. At this temperature, the polymer chains collapse due to rearrangement caused by the interruption of hydrogen bonds and dehydration. As a result of the temperature rise above the LCST, the shrinkage of the thermo-responsive segments of the polymer chains in the carrier allows the drug to diffuse out of the particles’ interior. The increase in tumor tissue temperature can also be caused by permanent inflammation in the tumor. The temperature-related stimulus can also be completed by the LCST-controlled chemical composition of the copolymer or polymer used to prepare the individual system.

The redox-potential-responsiveness is noticed via the breakage (chemical oxidation) of certain bonds, including disulfide, ditelluride, aminoacrylate, borane, and oxalate, in the presence of a redox agent—GSH, naturally present in cancer tissue. For instance, disulfides are reduced to thiol groups by GSH [19], at high (2–10 mM) concentrations in tumor intracellular space [20]. In turn, the reduction of the ditelluride linkage (-Te-Te-) in the presence of GSH yields ditelluride hydroxyl [21]. As a result of the linkage reduction, the polymer chain fragmentation occurs, releasing the drug.

Exposure of the drug-carrying particles and decorated Fe_3_O_4_ to a high-frequency magnetic field induces local inductive heating in the cancer tissue, and consequently, the temperature increases by several dozen degrees. For instance, in the case of liposomes (LPs) containing magnetic NPs, under a high-frequency magnetic field, the temperature of the incorporated drug (doxorubicin) increased gradually from 37 °C to 57 °C [22]. The heating of the liposomal microparticle affected the lipid bilayer to melt, leading to local drug release at the target site.

It is worth adding that a similar result—the decay of particles (particle decomposition) under the influence of temperature—also occurs under factors such as photoacoustic waves, ultrasound, near-infrared light, radio-frequency, microwave, and a high-intensity focused ultrasound, initiating the release of the drug from the decomposed particles [22]. Especially LPs and nano- and microparticles containing lipids are prone to decomposition at elevated temperatures.

In turn, photosensitizers, light with wavelengths in the range 700–800 nm, and oxygen are used in photodynamic therapy (clinically approved for cancer therapy), which induces damage to cancer cells, leading to their death. Photosensitizers are compounds that, at certain wavelengths of light, are excited to produce singlet oxygen. It is worth knowing that two types of photosensitizers have been elaborated on. In the first type, excited photosensitizers generate oxygen-containing radicals, such as O_2_-·, HO·, and H_2_O_2_, via charge transfer, whereas photosensitizers of the second type produce singlet oxygen via energy transfer. As a result, the continuously produced reactive oxygen species are consumed by cancer cells, causing their oxidative burst [23]. Photodynamic therapy is usually combined with chemotherapy, i.e., photosensitizers and anti-cancer drugs are in the same particle, resulting in synergistic antitumor efficacy.

The ultrasound is used to execute one of three known effects: heat, acoustic cavitation, and acoustic radiation [24]. The growth, oscillation, and collapse of small gas bubbles under the influence of a varying pressure field of a sound wave in a fluid (aqueous) medium is known as acoustic cavitation. Thanks to acoustic cavitation, micro-pores are formed in the walls of blood vessels, which can be penetrated by ultrasound-sensitive particles carrying drugs.

Moreover, the increased ultrasound deposited in the body affects tumor ablation. In the case of acoustic cavitation, the generated bubbles increase absorption and acoustic energy, causing local stress and extravasation of cell.

The high-intensity focused ultrasound-mediated DDS system delivers anti-cancer drugs to the targeted tumor tissue non-invasively. The ultrasound source is located far from the irradiated tissue, and the beams are collimated into a millimeter-sized spot. It can penetrate to a relatively great depth. Thanks to the ultrasound beams, the temperature of the local tissue is raised, but maintained low enough to protect the tissues. However, local heat increases the membrane permeability, delivering anticancer drugs [25,26,27,28].

The ultrasound-induced heat may act on polymer or lipid chains in various ways [29]. For instance, in the lipid-bilayer particles containing silica shell known as cerasomes, the alkyl chains of 1,2-dipalmitoyl-sn-glycero-3-phosphocholine (DPPC) undergo rearrangement, leading to an increase in the membrane volume, whereas 1,2-distearoyl-sn-glycero-3-phosphoethanolamine-N-[methoxy(polyethylene glycol)-2000] (DSPE-PEG2000) influences membrane destabilization, followed by generation of the pores. These events increase the permeability of cerasomes’ bilayers by forming small gas nuclei that collapse on lipophilic segments of chains, leading to drug release [29].

There are also DDSs in which release occurs directly at the tumor with enzymatic cleavage of chemical bonds between the prodrug and polymer carrier. The enzymes occur naturally at high concentrations in cancer tissue, unlike their quantity in healthy organs.

An overview of references describing stimulus-sensitive DDSs is presented in Table 1.

## 3. Chemical Structures of Stimulus-Sensitive Drug Delivery Systems

The nano- and microparticles designed for stimulus-sensitive systems belong to various chemical groups and may contain hierarchical structures. The majority of the particles are produced in the way of synthesis, only a few natural materials can be used almost directly in preparation the final delivery systems.

The synthesis of polymers/copolymers or complex systems requires multi-stage, long-lasting processes. Here, we present examples of copolymers and complex materials used for polymer nano- or microparticles in stimuli-sensitive applications and briefly describe the source compounds.

### 3.1. Chemical Structures of the pH-Stimuli-Responsive Materials

**Figure 2:**
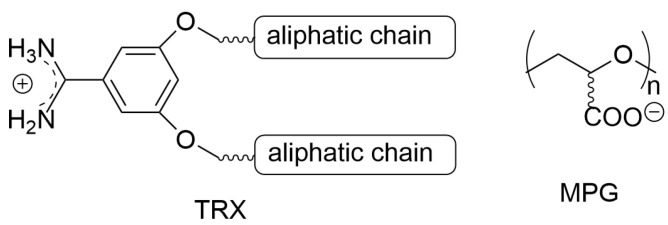
Chemical structures of 3,5-dipentadecyloxybenzamidine (TRX) and carboxyl-modified polyglycidol (MPG) for liposomal particle preparation (based on drawing from Ref. [67]).

The pH-sensitive liposome carrier containing an interferon–gene lipoplex (IFN-encoding plasmid DNA) was applied to murine dendritic cell line for induction of IFN-γ protein production [67]. The polymer-modified liposome particles (hybrid complex) consisted of IFN-encoding plasmid DNA loaded into a lipoplex containing a benzamidine aliphatic derivative, decorated with hydrophilic polyglycidol derivative (MPG). The 3,5-dipentadecyloxybenzamidine hydrochloride (TRX) was commercially available, while the polyglycidol, with a degree of polymerization (DP) of 76, was synthesized from glycidol, and subsequently, the hydroxyls underwent modification to obtain carboxyl anions in each repeating unit (see Figure 2).

**Figure 3:**
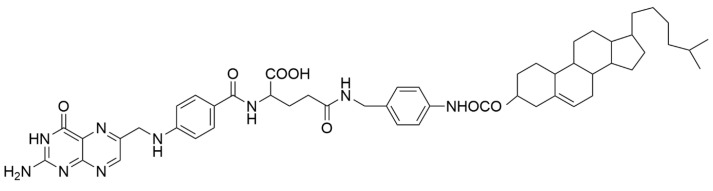
The product of synthesis described in Ref. [70].

The preparation of cholesterol-p-aminobenzylamine-folate consisted of the synthesis of cholesterol chloroformate with p-aminobenzylamine and subsequent conjugation with folic acid [70]. The product of synthesis (presented in Figure 3) was used in the preparation of LPs loaded with an anti-cancer drug.

A pH-sensitive DDS containing ester bonds able to hydrolyze at an acidic pH in the extracellular space of a tumor was developed by Yang et al. [77].

**Figure 4:**
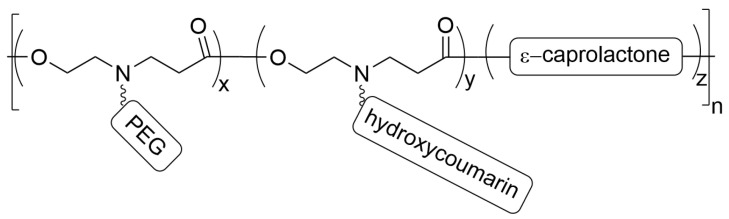
Conceptual structure of mPEG-b-HCou-g-MPCL copolymer from Ref. [77].

The authors prepared pH-sensitive fluorescent micelles (MCs) composed of a diblock copolymer of ε-caprolactone and 3-((2-hydroxyethyl)(prop-2-yn-1-yl)amino)propanoate-grafted mPEG2000 and 7-hydroxycoumarin (HCou) using click chemistry, presented in Figure 4.

**Figure 5:**
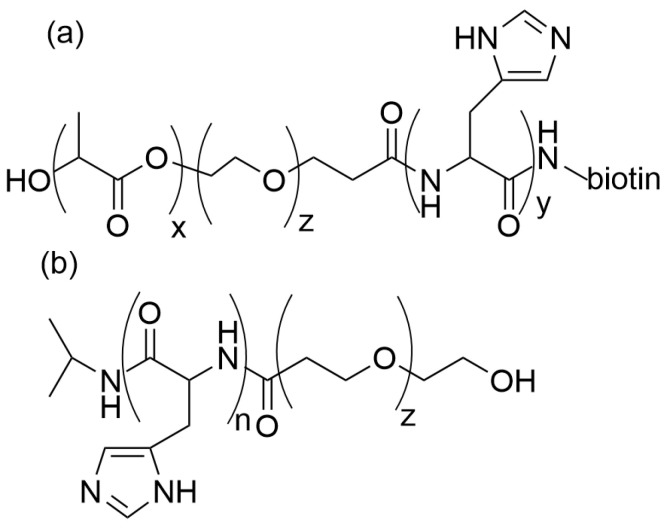
Chemical structures of (**a**) pLLA-b-PEG-b-polyHis-biotin and (**b**) polyHis-b-PEG copolymers presented in Ref. [80]. Reprinted with permission from the Ref. [80].

In turn, Lee et al. [80] synthesized pH-sensitive triblock copolymer poly(L,L-lactide)-b-PEG-b-poly(histidine-biotin) and admixed it with polyhistidine-b-PEG (polyHis-b-PEG) to form mixed MCs (see Figure 5a,b). L-Histidine is an amino acid responsible for the buffering of biological systems. Its base structure has pK_b_ = 6.5. At pH above 7, the polyHis-biotin is hidden in the screened PEG-containing micelle shells. In the tumor environment, at a pH of approximately 6.5, the polyHis carrying biotin exposes the copolymer chains with biotin on their ends to the micelle surface, allowing binding to tumor cell receptors. When the pH is below 6.5, the MCs undergo decay and release the drug.

The oligosaccharides also served as pH-sensitive polymer carriers, according to a procedure developed by Manchun et al. [87]. To obtain pH-reactive NGs, the authors used commercially available dextrin (molecular weight 1400) crosslinked with formaldehyde. The system remained stable at pH > 7, whereas in acidic environments, such as those found in tumors, the acetal bond was cleaved, releasing the biologically active compound.

### 3.2. Chemical Structures of Temperature-Sensitive Carriers

The induced local hyperthermia acts on the delivery particles in the tumor, releasing the drug immediately upon the stimulus. The LPs and thermosensitive polymers are chemically modified to develop drug carriers.

Motamarry et al. prepared lysolipids from hydrolyzed lipids to manufacture thermosensitive LPs that can release an anti-cancer drug at hyperthermia (40 °C) [97]. Soft hyperthermia is induced locally by high-intensity focused ultrasound. The LPs were obtained from the mixture of monostearoyl-2-hydroxy-sn-glycero-3-phosphocholine (MSPC), 1,2-dipalmitoyl-sn-glycero-3-phosphocholine (DPPC), and 1,2-distearoyl-sn-glycero-3-phosphoethanolamine-N-[methoxy (polyethylene glycol)2000] (DSPEPEG2000). The identical lipid composition for the thermo-sensitive LPs was used by Lyu et al. [98].

The release of a thermo-triggered drug from copolymer MCs decorated with a folate acid was carried out by Pluronic F127-poly(D,L-lactic acid) (F127-PLA) [95]. The polymerization degree of poly(D,L-lactic acid) was equal to 100. Pluronic F127 is a commercially available triblock copolymer containing poly(propylene oxide) as the central block and poly(ethylene glycol) as side blocks, with a total average molecular weight of 12,600 g/mol. The F127-PLA block copolymer was obtained by ring-opening polymerization of PLA on the hydroxyl group of Pluronic F127, initiated by tin octanoate. The second end of poly(ethylene glycol) of Pluronic was folic acid-modified using N-hydroxysuccinimide and 1,1′-carbonyldiimidazole hydrochloride as a linker to form an ester bond.

**Figure 6:**
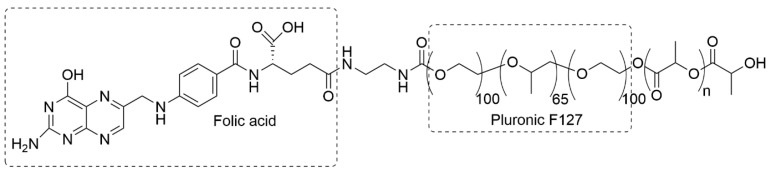
Chemical structure of F127-PLA carrying folic acid (based on Ref. [95]). Reprinted with permission from the Ref. [95].

It was found that at 37 °C, the copolymer MCs were stable, whereas at nearly 40 °C, the drug was released due to micelle shrinkage. This was because the copolymer exhibited a low critical solubility temperature of 39.2 °C and underwent dehydration above that temperature. The chemical structure of the copolymer F127-PLA with covalently attached folic acid is presented in Figure 6.

It is known that polypeptides reversibly associate during heating. The critical temperature for forming associates depends on the polymer’s hydrophobicity, molecular weight, and concentration [100]. When the peptide is heated, it may adopt an α-helical structure, which enables aggregation and the formation of larger particles. The transition of the peptide conformation must occur around 40 °C, which is the goal of engineering polypeptides for drug delivery with a thermal stimulus. Being thermosensitive, elastin-like polypeptides (ELPs) are especially useful because they self-associate and phase-separate in response to heating. They consist of repeating (aPGbG)_n_ where the guest residue, b, can be any amino acid except proline, and a is valine or isoleucine [215]. The temperature of the ELP transition depends on the guest residues (a and b), concentration, molecular weight, and ionic strength (NaCl concentration). A study of circular dichroism showed that ELP forms type II β-turns. This form of ELP undergoes a phase transition within a temperature window of less than 2 °C [216].

**Figure 7:**
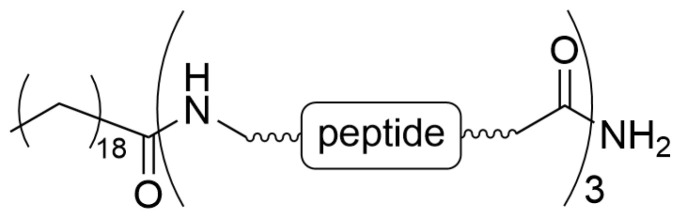
The conceptual structure of the modified peptide described in Ref. [26].

Park et al. elaborated on the LPs combined with elastin-like polypeptides to obtain a system that, upon high-intensity focused ultrasound, released the drug [26]. The particles were composed of 1,2-dipalmitoyl-sn-glycero-3-phosphocholine (DPPC), 1,2-distearoyl-sn-glycero-3-phosphoethanolamine-N-[methoxy(polyethyleneglycol)-2000] (DSPE-PEG-2000), cholesterol, and a fatty acid-conjugated elastin-like polypeptide (ELP). Cholesterol decreases the fluidity of the liposomal bilayer at physiological temperatures and limits drug leakage from the particle interior. The N-group of ELP was conjugated with a single stearyl group (C18) to anchor the lipid bilayer in the liposome, and the C-terminal group was amidated to control the carrier solubility. The chemical structure of the modified elastin-like polypeptide is presented in Figure 7. The particles obtained were more stable and remained in the blood longer than circulating conventional LPs.

A different thermosensitive-particle-carrier approach was proposed by Li et al., who developed solid and hollow gold NPs incorporated into LPs [107]. Using gold NPs, one can precisely tune the transition temperature of liposomes to the physiological conditions. The transition temperature of the gold-free LPs was 60 °C, whereas the transition temperature of the composite LPs decreased to the desired 41 °C.

In another study, the hydrogel made of Pluronic F127 served as the thermosensitive agent in the incorporated NPs [112]. The particles were composed of the cationic surfactant N-[1-(2,3-dioleoyloxy)propyl]-N,N,N-trimethylammonium methyl sulfate (DOTAP) and monomethoxy poly(ethylene glycol)-b-poly(ε-caprolactone) diblock copolymer with M_n_ = 4000. The thermosensitive Pluronic F127 formed hydrogel gels above 25 °C, remaining in its liquid state below room temperature. Thanks to the complex system incorporated into the hydrogel, the hydrophobic drug remained in the target tissue for a longer time and at a higher concentration.

### 3.3. Chemical Structures in Redox Stimulus Carriers

**Figure 8:**
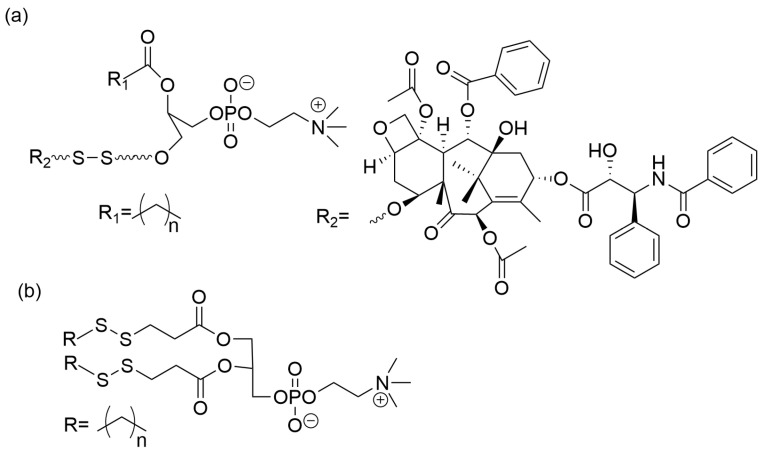
Chemical structures of (**a**) PTX-ss-PC described in Ref. [13]; (**b**) di-3-(dodecyldisulfanyl)propyl phosphatidylcholine described in Ref. [11]. Reprinted with permission from the Ref. [11].

Wang et al. developed a redox-responsive system based on disulfide –S-S- reduction and drug liberation at the target site [13]. The disulfide bond between Paclitaxel and a lysophosphatidylcholine prodrug (PTX-SS-PC) was reduced, forming LPs. The compound was synthesized using a five-step conjugation method that started with 1-stearoyl-2-hydroxy-sn-glycero-3-phosphocholine and paclitaxel with a hydroxyl group blocked with t-butyldimethyl chlorosilane. The structure of PTX-SS-PC is presented in Figure 8a.

In another study, the –SS- was included in a phosphatidylcholine derivative to yield di-3-(dodecyldisulfanyl)propyl phosphatidylcholine [11]. The compound was synthesized in sequence reactions, starting with 3-(tritylthio)-propanoic acid and L-α-glycerophosphorylcholine. In the following step 2-(dodecyldisulfanyl)pyridine yielded the final phosphatidylcholine derivative with –SS- bonds. The chemical structure of the redox component of LPs is presented in Figure 8b.

**Figure 9:**
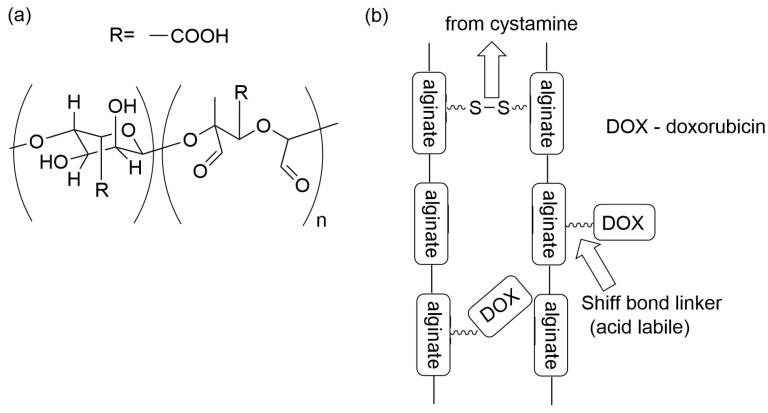
Chemical structure of (**a**) modified alginate polymer and (**b**) crosslinker of alginate chains containing –SS- bond described in Ref. [114].

In turn, the –SS- bridge was introduced to crosslink alginate chains of the alginate derivative, forming particles [114]. For that purpose, the hydroxyls of alginate rings were oxidized, followed by crosslinking with cystamine dihydrochloride. Simultaneously, in the following step, the doxorubicin hydrochloride was bound covalently via an acid-labile Schiff bond linker and via ion-exchange reaction of negatively charged carboxyl groups with positively charged doxorubicin molecule (see Figure 9).

**Figure 10:**
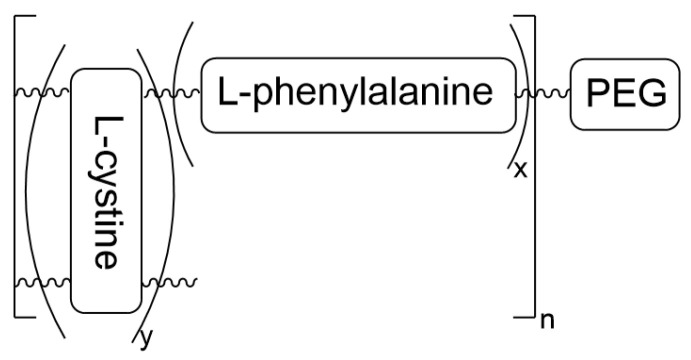
Conceptual structure of methoxy-poly(ethylene glycol)-poly(L-phenylalanine-co-L-cystine) described in Ref. [10].

The –SS- crosslinker from the L-cystine derivative of polypeptide nanoformulation was applied to be cleaved in the tumor environment. The reductive stimulus acted on the –SS- bond, resulting in the release from the methoxy-poly(ethylene glycol)-poly(L-phenylalanine-co-L-cystine) NGs composed of a combined small dose of doxorubicin with 1-methyl-D,L-tryptophan to suppress the tumor growth [10]. The chemical structure of the copolymer forming NGs is presented in Figure 10.

Among other than –SS- crosslinkers, the diselenium bond undergoes cleavage much more easily than the disulfide because the selenium atom is bigger, and hence, the energy of the bond (equal 172 kJmol^−1^) is lower than the –SS- bond energy (equal to 240 kJmol^−1^) [4]. Thus, the diselenide bond is more prone to oxidation than the disulfide in mild conditions. This behavior enables the use of diselenium bonds in polymer carriers for controlled drug release in response to an external stimulus.

**Figure 11:**
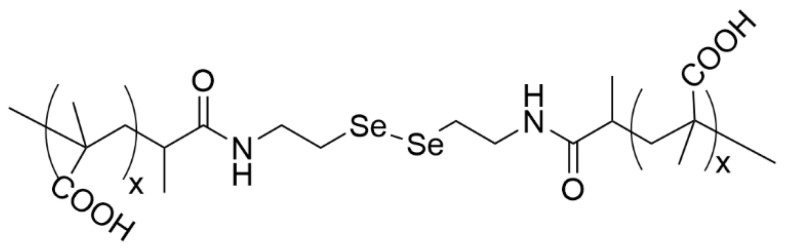
Conceptual structure of the poly(methacrylic acid) with diselenide crosslinker described in Ref. [4]. Reprinted with permission from the Ref. [4].

Tian et al. prepared NGs via the distillation–precipitation copolymerization of methacrylic acid (MAA) and a diselenide cross-linker (shown in Figure 11), N,N′ -bis(methacryloyl) selenocystamine (BMASC), using 2,2-azobisisobutyronitrile (AIBN) as the initiator [4].

**Figure 12:**
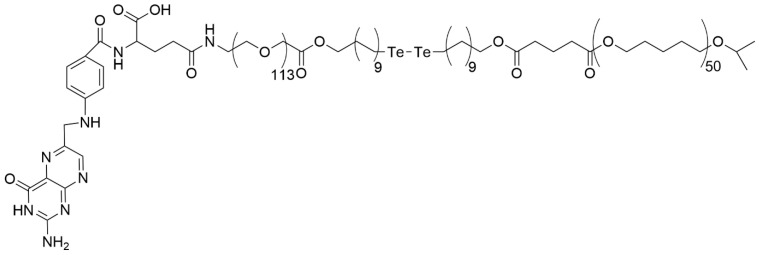
Chemical structure of copolymer containing ditelluride linkage described in Ref. [21].

The ditelluride crosslinker was used in the fabrication of the poly(ε-caprolactone)-PEG copolymer-ended folic acid. The crosslinker was incorporated into the folic acid-ended PEG through a coupling reaction. Then, poly(ε-caprolactone) ended with a carboxyl group was synthesized via anionic polymerization using aluminum isopropoxide as an initiator and combined with PEG carrying folic acid and a ditelluride hydroxyl-ended alkyl group (see Figure 12) [21]. The copolymer was a substrate for the preparation of doxorubicin-loaded MCs.

The binding energy of the ditelluride bond, equal to 149 kJ/mol, is lower than that of diselenide and disulfide; thus, the ditelluride in the polymer drug carrier is the most reactive of the three mentioned compounds, suitable for a reductive stimulus carrier [217].

### 3.4. Chemical Structures Used in Magnetic Field Stimulus Carriers

When ferromagnetic particles are exposed to a high-frequency magnetic field, they are moved (to the target site) and simultaneously heated to a certain temperature above body temperature, even up to 56 °C.

The ferromagnetic NPs belong to anticancer drug carriers designed to respond to the stimulus of a magnetic field and are usually located inside lipophilic or polymer particles or embedded into the surface of the carrier.

To obtain a magnetic-field-sensitive carrier, the superparamagnetic particles (gamma-ferric oxide NPs) were embedded into LPs [128]. The synthesis of magnetic particles (described in detail in Ref. [218]) was composed of the alkaline coprecipitation of ferric and ferrous chloride salts upon adding NH_4_OH (24–28%) aqueous solution at pH = 10 and at a temperature 80°C under vigorous stirring of the mixture. The heating of the mixture was maintained for 30 min. more. In the final step, the magnetite suspension was purified through multiple washes with water. Babincová et al. stated that phospholipids have a strong affinity for ferrocolloids suspended in chloroform, which leads to the adsorption of phospholipids onto the magnetic particles and the formation of a nanoparticle anchoring in the phospholipids [128].

Hardiansyah et al. elaborated on the PEGylated LPs with citric-acid-coated magnetic particles [219]. Due to the citric acid adsorbed on the surface of magnetic NPs and thus making their surface hydrophilic, the ferrocolloids were embedded in the PEGylated—hydrophilic—shell of the LPs [22]. The PEG in the particle’s corona assured the biocompatibility and stability of the particles.

In turn, Yang et al. developed a method for producing metal-organic Fe_3_O_4_/ZIF-8-Au_25_ nanocrystals for magnetic targeted anticancer therapy [130]. The composed NPs were prepared from an aqueous solution containing a mixture of Fe_3_O_4_ nanocrystals and methylimidazole (MeIM), followed by the addition of Zn(NO_3_)_2_ in water using a syringe pump. This procedure yields Fe_3_O_4_/ZIF-8 NPs. In the final step, the nanoclusters of the Au_25_(Capt)_18_ were attached to the Fe_3_O_4_/ZIF by a ligand exchange reaction. Under NIR light irradiation, Au clusters produce singlet oxygen (^1^O_2_) to destroy abnormal cancer cells in consequence of photodynamic therapy.

The magnetic NPs can be placed in the core of core-shell particles, of which the shell is composed of oligosaccharides, like gum Arabic [133], an ethylene diamine-derivative of β-cyclodextrins [151], LPs [134], liposome microbubbles [147], PEGylated LPs [134], mesoporous silica [137], or amphiphilic copolymers like poly(ethylene glycol-b-ε-caprolactone) [136].

The liposome microbubbles were fabricated in a few-step process. First, the ferromagnetic LPs were formed using the classical method of embedding the ferromagnetic NPs and the anti-cancer drug into liposomes. The LPs were composed of 1,2-distearoyl-sn-glycero-3-phosphoethanolamine-N-[maleimide(polyethylene glycol)-2000] (DSPE-PEG2000-MAL), 1,2-distearoyl-sn-glycero-3-phosphocholine (DSPC), 1,2-distearoyl-sn-glycero-3-phosphoethanolamine-N-[methoxy (polyethylene glycol)-2000] (DSPE-MPEG), and 1,2-distearoyl-sn-glycero-3-phosphoethanolamine-N-[PDP(polyethylene glycol)-2000] (DSPEPEG2000-SPDP) and cholesterol. Then, the thiol-activated microbubbles were prepared using DSPE-PEG2000-SPDP in a solution of propylene glycol, glycerin, and water (1:1:8) with perfluorocarbon gas (C_3_F_8_, PFC). The disulfide bond in the N-succinimidyl 3-(2-pyridyldithio)propionate (SPDP) end group of DSPE-PEG2000 was cleaved by dithiothreitol. The PFC gas served as a bubbling agent of the liposome mixture. Shaking the solution vigorously at high speed and group exchange via sulhydryl bonds yielded functionalized microbubbles with covalently attached LPs carrying the drug [147]. The studies revealed that microbubbles were prone to magnetic fields and ultrasound pulses. Upon permanent exposure to ultrasound pulses, the drug was instantly released.

It is known that Fe_3_O_4_ NPs, due to their capability to aggregate and oxidize in a biological environment, undergo chemical modification of their surface. The hydrophobic surface and instability in aqueous media of magnetic NPs often limit their biomedical application in drug delivery carriers. For that reason, the magnetite NPs are coated with polymers or surfactants suitable for aqueous conditions. Hong et al. fabricated polypyrrole-coated magnetic nanoparticles containing grafts of ethylenediamine derivative of β-cyclodextrin with hyaluronan acting as an intermediate linker [151]. First, the surface of Fe_3_O_4_ NPs was modified by sodium dodecyl sulfate. Then, the shell was formed on the NPs’ surfaces, copolymerizing pyrol, FeCl_3_·6H_2_O, and hyaluronic acid added to the suspension of the NPs. The dry hyaluronic acid-modified particles were decorated with β-cyclodextrins at the surface using 3-[3-dimethylaminopropyl]carbodiimide hydrochloride (EDC) and N-hydroxysuccinimide (NHS). The EDC and NHS are used for the activation of carboxyl-containing hyaluronic acid in the esterification process with amine-substituted β-cyclodextrins.

### 3.5. Chemical Structures of Light-Sensitive Carriers

The light can act on light-stimuli carriers in various ways.

The light-sensitive anticancer nano- and microparticles contain, among others, metal NPs such as gold nanorods, nanoshells, and nanocages, which generate a photothermal response resulting in the hyperthermic killing of cancer cells [153]. Moreover, the amphiphilic copolymers may contain a photoactive agent (as a photosensitizer) that undergoes photo-induced cleavage upon light irradiation. The photosensitizer can also be covalently attached to the phospholipids, forming the LPs. In this case, upon exposure to NIR, the photosensitizer (i.e., phthalocyanine derivative) becomes hydrophobic due to a conformational change. The hydrophobic form interacts with cancer cells’ membranes, leading to their breakage [167]. The light irradiation process of the particles carrying an anticancer drug results in decay (collapse) of the copolymer of the delivered nanoparticle, and the block of the copolymer itself can affect tumor cell survival. The light can also generate singlet oxygen (^1^O_2_), which leads to the production of clean by-products. In turn, the increased oxygen levels promote the formation of singlet oxygen, which drives greater oxygenation of the cancer tissue and strengthens the ability to fight cancer.

Huang et al. presented gold nanorods to a light-sensitive (near-IR) stimulus delivery system for an anticancer drug. The gold nanorods were produced according to the method described in Ref. [220]. Briefly, the method of gold nanorod synthesis consisted of two steps: preparation of “seed” and “grow” solutions. In the first step, the so-called “seed” solution was obtained. It consisted of mixing hexadecyltrimethylammonium bromide (CTAB) with HAuCl_4_ at 25 °C, followed by the addition of the reducing agent NaBH_4_, which yielded a brown-yellow color indicating the formation of NPs serving as “the seeds”. In the second step, the CTAB solution was mixed with AgNO_3_ at 25°C. Then, HAuCl_4_ was added to the stirred solution and, after a while, ascorbic acid. Under the influence of this mild reducing agent, the mixture’s dark yellow color becomes transparent. Finally, the small portion of the “seed” solution and the “grow” solution was admixed at an elevated temperature (ca. 30 °C), yielding gold nanorods. Using the procedure described above, gold nanorods with aspect ratios of 1.5–10 can be synthesized, it was found that CTAB-gold nanorods were very stable in human serum-containing media.

**Figure 13:**
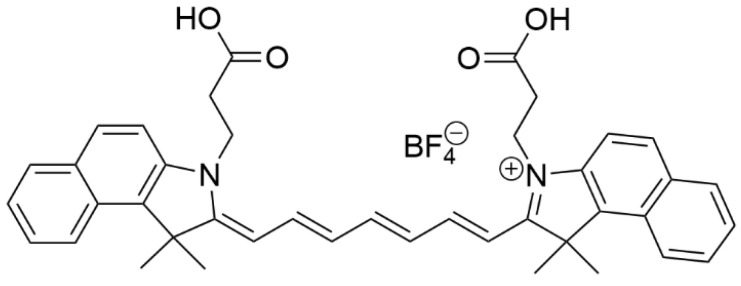
Chemical structure of cypate dye.

To generate local heat, organic near-infrared (NIR) cyanine dyes such as indocyanine green and cypate are embedded in LPs, together with NH_4_HCO_3_ and an anticancer drug (as shown in Figure 13). The energy produced by excitation of the NIR cyanine dye generates a local increase in temperature (ca. 42 °C), which leads to the thermal decomposition of the inorganic salts such as NH_4_HCO_3_ into CO_2_ bubbles. In turn, it destroys the LPs’ bilayer [155].

**Figure 14:**
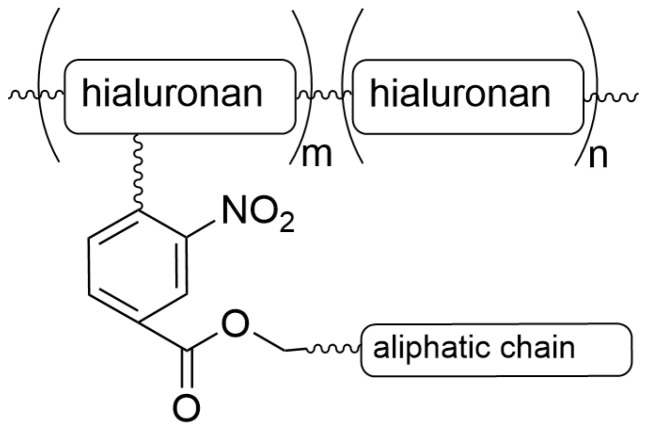
The conceptual structure of the HA-NB-SC copolymer used for the preparation of particles. Based on Ref. [156].

Sun et al. elaborated on MCs composed of a hyaluronan-o-nitrobenzyl-stearyl (HA-NB-SC) copolymer, which decomposes upon short UV irradiation at 365 nm [156]. UV irradiation results in a cleavage of the copolymer, leading to a nitrosobenzaldehyde derivative, followed by decomposition of the particles in the target. The incorporated drug interacts with cancer cells, inhibiting their proliferation [221]. The ortho-nitrobenzyl group serves as a photo-cleavable labile linker. The amphiphilic copolymer of HA-NB-SC was synthesized in a few steps. First, o-nitrobenzyl-stearyl was obtained from 3-nitro-4-(bromomethyl)benzoic acid, which was esterified with stearyl alcohol in the presence of N,N’-dicyclohexyl carbodiimide and catalytic 4-dimethylaminopyridine. Then, the hyaluronic acid was mixed with the NB-SC oligomer in dried dimethyl sulfoxide at 40 °C for 48 h and purified via multi-step dialysis [156]. The HA-NB-SC is presented in Figure 14.

**Figure 15:**
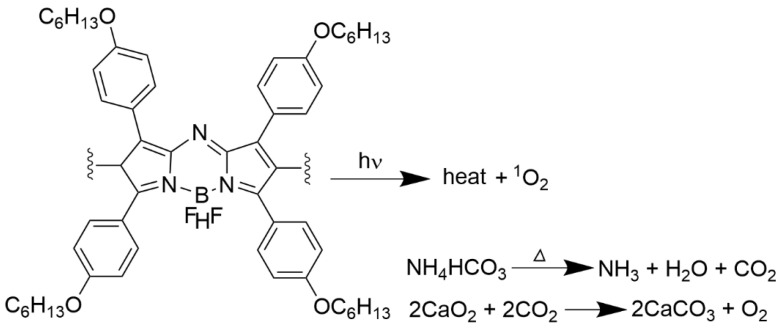
Chemical structure of aza-BODIPY derivative described in Ref. [157].

The photo-sensitizer 4,4-difluoro-4-bora-3a,4a-diaza-s-indacene, known as an aza-BODIPY derivative with four alkil chains (as presented in Figure 15), was used in the PEGylated LPs together with NH_4_HCO_3_ and CaO_2_ NPs, both embedded in the LPs interior space [157]. In addition to local hyperthermia upon NIR, the particles irradiation leads to melting of the LPs, resulting in a gradual increase in the oxygen concentration due to the production of the singlet oxygen from aza-BODIPY heating. The addition of CaO_2_ to NH_4_HCO_3_, which served as an oxygen supplement, assured the stable delivery of oxygen to cancer cells.

In another work, the collective action of aza dye–manganese (II) phthalocyanine magnetofluorescent carbon dot-containing PEGylated LPs was applied to produce oxygen species against tumor growth [159]. It was found that in consequence of NIR (at λ = 745 nm), ^1^O_2_ is produced, which catalyzes the decay of H_2_O_2_ to H_2_O and O_2_.

**Figure 16:**
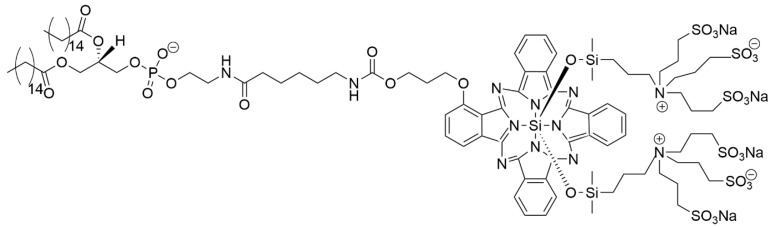
IR700 with bound 1,2-dipalmitoyl-sn-glycero-3-phosphoethanolamine (DPPE) (described in Ref. [167]).

Kono et al. found that photosensitizer IR700, a silicon phthalocyanine derivative conjugated to antibodies, breaks the liposome membrane upon exposure to NIR [167]. The synthesis of 1,2-dipalmitoyl-sn-glycero-3-phosphoethanolamine (DPPE) with incorporated IR700 yielded a product for the construction of NIR-sensitive LPs for drug delivery. The IRDye700DX NHS ester was conjugated with DPPE. The modified IR700 was attached to DPPE using trimethylamine as a linker. Figure 16 presents IR700-conjugated DPPE.

### 3.6. Chemical Structures of Ultrasound-Sensitive Carriers

For ultrasound-sensitive carriers, various materials have been used, like silica [222], gold, polymeric, and liposomal NPs. The NPs attached to the bubbles can be cooperatively injected into the region of interest.

There are various combinations of bubbles with anticancer DDSs. For instance, Xin et al. elaborated on an ultrasound-sensitive system composed of PLGA NPs carrying an anti-cancer drug embedded into liposomal bubbles [176]. If the liposomal bubbles are exposed to ultrasound at the target site, the PLGA NPs produce vibrations that break the liposomal membrane, releasing the PLGA NPs. The LPs were prepared from the mixture of 1-palmitoyl-2-stearoyl-sn-glycero-3-phosphatidylcholine (HSPC), 1,2-distearoyl-sn-glycero-3-phosphocholine (DSPC), and 1,2-distearoyl-sn-glycero-3-phosphoethanolamine-N-[methoxy(polyethylene glycol)-2000] (DSPE-PEG2000), and the PLGA NPs were produced from commercial poly(D,L-lactic-co-glycolic acid) PLGA (50:50), with a M_w_ = 15 kDa.

**Figure 17:**
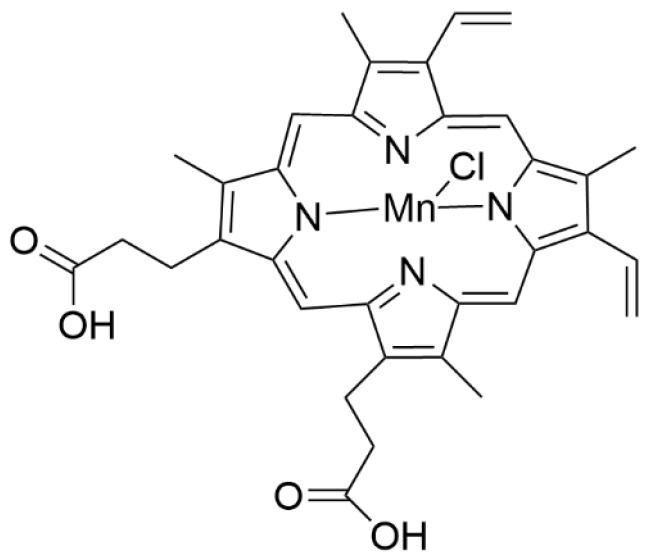
The Mn(III) protoporphyrin IX chloride described in Ref. [177].

In another study, a manganese protoporphyrin was incorporated into the folate-containing interior of LPs [177]. When LPs in cancer tissue were exposed to ultrasound irradiation, singlet oxygen was generated due to manganese’s ability to act in the protoporphyrin system. The Mn (III) protoporphyrin IX chloride is shown in Figure 17.

In turn, the anticancer drug can be effectively released from nanodroplets containing the perfluoropentan core and poly(ethylene glycol)-b-poly(ε-caprolactone) (PEO-PCL) shell [179]. The nanodroplets formed via acoustic droplet vaporization are then converted into nanobubbles under the influence of ultrasound and, in effect, undergo acoustic cavitation.

A mixture of two types of three-block copolymers, namely Pluronic P123 and Pluronic F127, was used for the manufacturing of mixed MCs [180]. The mentioned Pluronics differ in length of PEG and PPO blocks and molecular weight, which is 5800 and 12,600, respectively. Pluronics are FDA-approved three-block amphiphilic PEG-PPO-PEG block copolymers, often used for the preparation of MCs for biomedical applications. Under the influence of ultrasound-increased applied power, the MCs contract, expand, and finally explode, of which behavior leads to the stimulated release of the drug. In another study, a mixture of Pluronic P105 and PEG-diacylphosholipid was used for manufacturing of the stable MCs [181]. The PEG-diacylphospholipid–1,2-distearoyl-sn-glycero-3-phosphoethanolamine-N-[methoxy(polyethylene glycol)-2000 prevents the too fast destruction of MCs. It was found that when MCs were prepared exclusively from Pluronic, the drug release occurred due to the dissolution of the MCs below the critical micelle concentration in a biological environment (i.e., blood vessel) without any external stimulus. A mixture of lipids and their derivatives was used to prepare ultrasound-sensitive cerasomes composed of 1,2-dipalmitoyl-sn-glycero-3-phosphocholine (DPPC), 1,2-distearoyl-sn-glycero-3-phosphoethanolamine-N-[methoxy(polyethylene glycol)-2000] (DSPE-PEG-2000), and 1-stearoyl-2-hydroxy-sn-glycero-3-phosphocholine (MSPC) [29]. As a consequence of the ultrasound, the particles were heated to ca. 42 °C and partially melted. As a result of melting, the drug was rapidly released.

### 3.7. Chemical Structures in the Enzyme-Stimuli-Sensitive Systems

The enzyme-responsive systems belong to a new class of DDSs that release their cargo in response to pathological high levels of enzyme at the target site. The action of the drug carrier in this case is specific because of differences in the metabolism of healthy and cancerous tissue. Namely, in cancer cells, some enzymes are present in much higher amounts than in healthy cells. The drug carriers contain labile bonds in their chemical structure, prone to cleavage by enzymes upon reaching the tumor site. The enzymes involved in the cleavage belong to the hydrolase and oxidoreductase families. For instance, there are lipases (e.g., phospholipase A2, (PLA2)), proteases (e.g., metalloproteinases, cathepsins), glycosidases (e.g., β-mannanase), and oxidoreductases (e.g., dehydrogenase) [205].

**Figure 18:**
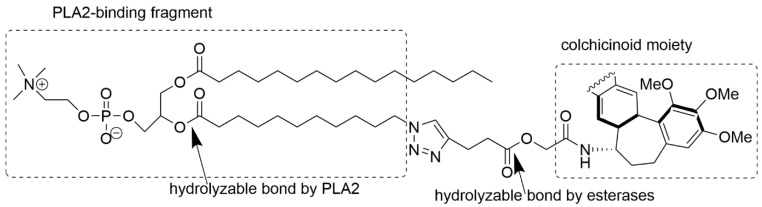
Chemical structure of colchicinoid-containing phospholipid described in Ref. [185]. Reprinted with permission from the Ref. [188].

The drug coatings can be constructed in various ways. For instance, the chemical structure of the “packaging” can contain ester-bound groups, which are labile and prone to hydrolysis. Shchegravina et al. constructed phospholipid-containing particles with an ester-hydrolyzable bond, which reacts with phospholipase A2 and covalently attaches a colchicinoid prodrug. The structural design of a colchicinoid-containing phospholipid is illustrated in Figure 18 [188].

The linker between phospholipid and colchicine was synthesized from azide containing 4-(dimethylamino)pyridine (4-DMAP), activated lyso-phosphatydylcholine, and functionalized with an allo-colchicinoid [188].

The syntheses of metalloproteinase-2 (MMP-2)-responsive LPs modified with the β-cyclodextrin were elaborated on by Ji et al. [189]. The surface of the LPs was equipped with a β-cyclodextrin sequence bound via a hydrophobic linkage, pirfenidone and gemcitabine as drugs, and modified tripeptide Arg-Gly-Asp. The β-cyclodextrin was exposed to the exterior from the liposome surface via the synthesized peptide CSSSGPLG-IAGQSSS. The sequence GPLG-IAGQ of the peptide is prone to cleavage by MMP-2 when the particle–drug complex is in cancer tissue. The peptide was cleaved in the tumor microenvironment by MMP-2. The cleavage was accompanied by the release of pirfenidone, which adjusts pancreatic stellate cells. In turn, the drug interacts with pancreatic tumor cells and stimulates local tumor growth. The gemcitabine released from the particles can recognize and kill pancreatic tumor cells.

Dorresteijn et al. elaborated on MMP-2-sensitive polylactide-b-polypeptide-b-polylactide three-block copolymer NPs [191]. The triblock copolymer was synthesized from lactide and difunctional polypetide via the anionic copolymerization of L-lactide. The MMP-2 specifically recognized and cleaved the Pro-Leu-Gly-Leu-Ala-Gly sequence within the polypeptide block.

The MMP2-sensitive system based on the hydrolysis of a gelatin-containing core and aminoPEG-shell quantum dot NPs coated with amino-PEG was prepared by Wong et al. [192]. The binding of amino-PEG to the NPs’ surfaces was performed using the 1-ethyl-3-[3-dimethylaminopropyl]carbodiimide hydrochloride (EDC)/sulfo-NHS coupling procedure. The authors designed the complex particles that can shrink in the tumor microenvironment. Thanks to the partial digestion of the NPs’ gelatin core by proteases (mostly MMP-2) present in the tumor tissue, the particles’ diameter decreased tenfold. The much smaller NPs can adjust their shape to the diameter of the blood vessel and penetrate tumor tissue, reaching the target site more effectively.

**Figure 19:**
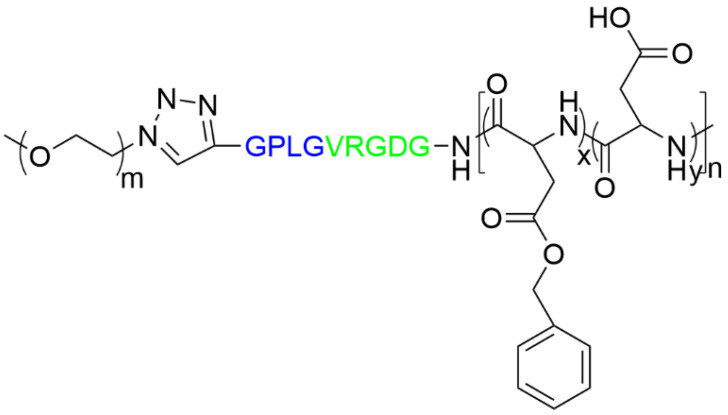
Chemical structure of a poly(β-benzyl L-aspartate) copolymer with partially removed benzyl side groups carrying an GPLGVRGDG oligopeptide and PEG. Reprinted with permission from the Ref. [193].

The polypeptide MCs prone to hydrolysis by the tumor peptidases were produced by Ke et al. [193]. Before particle preparation, the authors synthesized an oligopeptide with the amino acid sequence (-GPLGVRGDG-) and a covalently attached diblock copolymer composed of PEG and partially hydrolyzed poly(β-benzyl L-aspartate), as presented in Figure 19. The block copolymer was constructed using click chemistry to bind alkynyl-GPLGVRGDG to the end of PEG, which served as a macroinitiator in ring-opening copolymerization with β-benzyl L-aspartate N-carboxyanhydride (BLA-NCA) by terminal amino groups. In the final step, the copolymer carrying oligopeptide was subjected to the hydrolysis of poly(β-benzyl L-aspartate) units, which led to the partial removal of benzyl groups and improved the material’s hydrophilicity.

The mesoporous silica NPs decorated with gold NPs–biotin on the pore outlets at the NPs’ surfaces served as an enzyme-sensitive carrier of the anticancer drug [195]. The whole system was composed of mesoporous silica–peptide–gold NPs–biotin–doxorubicin. The gold nanoparticles–biotin conjugate acted as a cap with a metalloproteinase-cleavable (KDPLGVC) peptide linker. The mesoporous particles were synthesized using the basic catalysis sol-gel method [223]. The surface amine groups of the silica nanoparticles were modified to carboxyl groups using succinic anhydride. N-[6-(biotinamido)hexyl]-3′-(2′-pyridyldithio)-propionamide (biotin-HPDP) was used to attach to the surface of mesoporous silica. Before the attachment with silica–gold, biotin-HPDP was partially cleaved with tri-n-butylphosphine to obtain biotin-HP.

**Figure 20:**
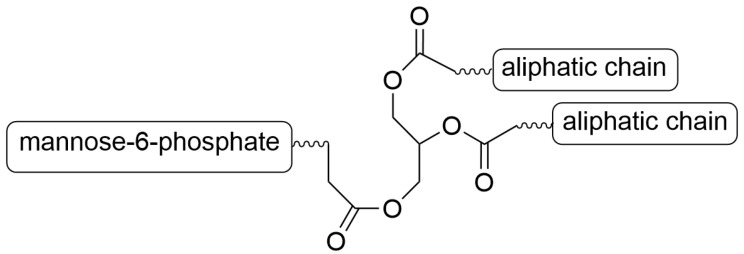
An amphiphilic phosphate lipid composed of mannose-6-phosphate, lipid part, a and three ester groups responding to two enzymes: esterase and phosphatase. Based on a drawing from the Ref. [194].

Mondal et al. constructed dual-enzyme-responsive MCs composed of an amphiphilic mannose-6-phosphate lipid, as shown in Figure 20 [194]. The synthesis of end-functionalized mannose-6-phosphate compounds was described in Ref. [224]. The advantage of the dual-responsiveness of MCs made from mannose-6-phosphate was that the MCs contained phosphate and ester groups, which are prone to hydrolysis by phosphatases and esterases. The enzymes are in lysosomes, and in MCs, they are unfolded. The important property of the micelle is that it is exclusively in the lysosomes of cancer cells.

Among oligosaccharides, a hyaluronic acid-grafted 2-aminoethyl methacrylate copolymer with hyaluronic acid-grafted lysine modified with 4-(4-(dimethylamino)phenyl-tetrazole)-benzoic acid (MTet) was applied to prepare a hyaluronidase-sensitive delivery system using microfluidics [197]. Hyaluronic acid-g-2-aminoethyl methacrylate (HA-g-AMA) was prepared through the amidation of HA and AMA in the presence of 4-(4,6-dimethoxy-1,3,5-triazin-2-yl)-4-methylmorpholinium chloride (DMTMM). In turn, HA-g-Lys-Mtet was prepared according to the recipe described in Ref. [225]. Hyaluronic acid grafted with a lysine segment was attached to MTet using N,N′-dicyclohexylcarbodiimide/4-dimethylaminopyridine.

**Figure 21:**
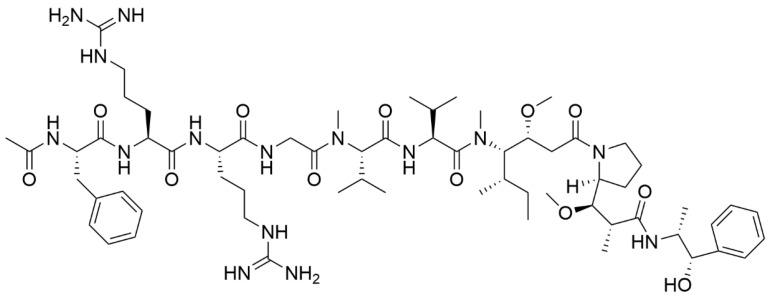
Monomethyl auristatin E is described in Ref. [203].

In turn, the cathepsin B-specific cleavable FRRG (Phe-Arg-Arg-Gly) peptide and tumor-specific monomethyl auristatin E were chemically conjugated in a one-step process (via DCCI/NHS method of amid bond formation) [203]. Arginine promotes particle penetration in tumor cells. The peptide was used as an enzyme-sensitive material. As a result of particle formation, stable and uniform NPs were prepared without a surface-active agent. The liberated monomethyl auristatin E (MMAE) in cancer cells inhibits tubulin polymerization, indicating that the tumor cells overexpress cathepsin B. The chemical structure of monomethyl auristatin E (FRRG-MMAE), which is specifically cleaved by cathepsin B, is presented in Figure 21.

**Figure 22:**
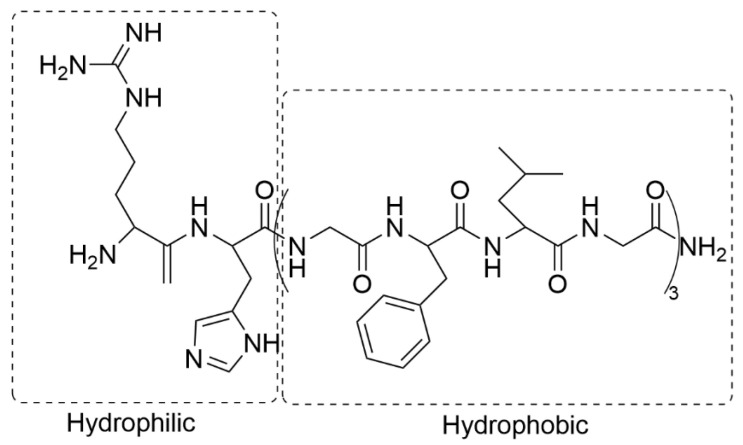
Chemical structure of enzyme-responsive oligopeptide composed of hydrophilic (corona) and hydrophobic (core) segments. The structure described in Ref. [204].

In similar studies, the particle-shell-forming peptide was composed of arginine and histidine segments combined with an enzyme-responsive oligopeptide (Gly-Lys-Phe-Gly)_3_ (GLFG)_3_, which formed the particle’s core [204]. The peptide responds to cathepsin B, favoring the controlled release of the drug in the cytoplasm. The chemical structure of the core-forming oligopeptide is presented in Figure 22.

## 4. The Methods of the Stimuli-Sensitive Carrier Preparation

The stimulus-sensitive carriers, although designed for a broad spectrum of stimuli, are manufactured using similar self-assembling methods. The recipes are adjusted to the particular carrier application, i.e., target organ of the body, carrier’s cargo, or material, from which the particles or carrier are made. The presented description of methods is very general, without particular nano- and microparticle production procedures. The more specialized methods are described in the individual literature reports.

The classical way for polymer nano- or microparticle preparation consists of (i) dissolving the copolymer (or polymer) and drug in the organic solvent and (ii) instillation of the mixture into water containing a surface-active agent to sterically stabilize the formed polymer chains’ self-assemblies with accompanying vigorous stirring. In the next step, the organic solvent–water mixture undergoes controlled-frequency sonication to unify and decrease the particle size. Finally, the particle suspension is left until the organic solvent evaporates or the mixture is dialyzed against water in dialysis bags with multiple exchanges for fresh water.

In the case of nano- or microparticles manufactured from carbohydrates, such as alginate derivatives, the polymer-modified oligosaccharides are dissolved in a water solution (usually buffer) with an activator of carboxyl groups (DCCI/NHS) and a crosslinking agent, i.e., cystamine dihydrochloride. Finally, the crosslinked particles are purified via dialysis [114].

In the case of the liposome formation, the mixture of phospholipids and/or phospholipids modified with hydrophilic polymers and cargo drug is dissolved in a volatile organic solvent. Then, the volatile solvent is evaporated (under vacuum), and the lipids are solidified on the glass walls, creating a thin film. In the final step, the glass vessel is filled with buffer solution containing the drug (i.e., protein), and the lipid sample is sonicated or subjected to several freeze–thaw cycles to produce a liposome suspension carrying the cargo. The excess of the unbound drug is removed via liposome dialysis against water. To standardize the liposome size, particles are extruded through a membrane with the desired size cut-off [30]. For hydrophobic drugs, the cholesterol is added during liposome formation to increase the drug concentration in the particle interior and stabilize the drug within the liposomal environment.

In turn, layer-by-layer (LbL) particles can be formed from template particles (such as charged polystyrene NPs), and a polymer with the opposite charge is added to obtain a two- or multilayer shell. When the NPs are polymer-coated, the unbound polymer is removed via multiple washes after each step [37].

An overview of the morphology of particles, predestined to stimulus-sensitive anticancer drug delivery, including corresponding references, is presented in Table 2.

## 5. Stimuli-Sensitive Systems in the Fight Against Individual Cancers

To combat a cancer of a particular internal organ, it often requires special treatment. The DDS suitable for curing the tumor in one organ may be inactive in fighting cancer in another, due to various reasons and circumstances. The whole drug system may be inappropriate to overcome a cancer because the drug release is too fast or too slow to achieve the therapeutic level (insufficient concentration). Alternatively, the chosen drug packaging and stimulus may be inappropriate to reach the target cells. Thus, the stimulus-sensitive system should be elaborated for each cancer separately.

Below are the described exemplary stimulus-sensitive systems that act on specific cancers.

### 5.1. Stimuli-Sensitive Carriers for Bladder Cancer Treatment

The stimuli-sensitive carriers for bladder cancer therapy are both LPs and polymers. The stimulus (e.g., heat or irradiation) source can be applied directly in the close vicinity of the tumor using a catheter. This section shows a short overview of the tested new strategies in fighting bladder cancer.

A pH-sensitive stearoyl-PEG-poly(methacryloyl sulfadimethoxine)-decorated liposome system was used to deliver protein to bladder cancer [30]. The system represents a proof-of-concept liposome system tested for drug delivery to the bladder epithelium. It was found that LPs at an acidic pH can adhere to epithelial cells. In this step, the LPs release their protein, and simultaneously, the payload interacts with macrophages involved in bladder cancer growth, inhibiting their action.

The phosphatidyldiglycerol-based thermosensitive LPs carrying doxorubicin (DPPG2-TSL-DOX) reinforced by hyperthermia applied by van Valenberg were used to effectively destroy bladder cancer in pigs [108]. The heat was generated using a urinary catheter combined with a radiofrequency-emitting antenna for 60 min. The results showed that doxorubicin toxicity in the body was significantly limited, and its concentration was highest in the bladder wall, with a homogenous distribution of the drug.

Similarly, tested on pigs, the lyso-thermosensitive liposomal (LTLD) doxorubicin system combined with hyperthermia was evaluated by Mikhail et al. [106]. The authors applied the LTLD commercial formulation ThermoDox^®^ (manufactured by Celsion Corp., Lawrenceville, NJ, USA), evaluated for hepatocellular carcinoma. The abovementioned formulation was administered intravenously, and mild hyperthermia was obtained via bladder irrigation with warm water. Using a liposomal system and hyperthermia, a synergistic cytotoxic therapy to bladder cancer was achieved.

In turn, Men et al. used catheter-administered positively charged complex polymer particles carrying a potential anticancer agent—deguelin [112]. The complex particles incorporated into a thermo-sensitive hydrogel of Pluronic F127 were described in Section 3.2. Thanks to the thermo-sensitivity of Pluronic and bioadhesive properties, the gelatinous system at higher (bladder) temperature released the drug for a longer time, maintaining its high local concentration in vitro and in mice. As a result, an anti-angiogenesis effect of daguelin in the tumor was registered.

### 5.2. Stimuli-Sensitive Carriers for Ovarian Cancer Treatment

Ovarian cancer is one of the common tumors in women, and the strategies to effective therapy are often chosen as the study subject. The selected stimulus-sensitive DDS for ovarian cancer treatment are presented below.

The broad use of some drugs, e.g., platinum-based anticancer chemotherapeutics, affects drug resistance by increasing metallothionein concentrations and GSH in tumor cells. In turn, the drugs are susceptible to degradation by tumor enzymes. For this reason, the drugs are sometimes modified to protect against enzymatic destruction. For instance, the modification of platinum (II) by introducing sterically hindered bulky ligands effectively allowed overcoming destruction by masking glutathione attack and additionally improved drug solubility [2]. This strategy was applied in the case of ovarian cancer treatment in the ovarian tumor human cell line ACP, resistant to drugs—a model—and compared to that based on human ovarian cancer cisplatin-sensitive A2780 [2]. The modified platinum in the form t,t-(pyridine)_2_Pt(NO_3_)_2_ was embedded into a copolymer carrier composed of a polyglycolic acid-forming core and PEG as a shell of the NPs. The study showed that the trans-geometry platinum (II) drug loaded in the copolymer carrier was able to successfully overcome drug resistance in human cell line.

Other therapies include LbL particles and NGs. The examples of other drug carriers used in therapy of ovarian cancer are presented below.

A pH-sensitive LbL particle made of polyamine and hyaluronan targets the CD44 receptor on ovarian (and breast) cancer stem cells and delivers a biomarker [37]. The tumor’s hypoxic, pH-induced structural reorganization of hyaluronan-containing NPs occurs due to electrostatic interactions between hyaluronan and polyamine. Additionally, the behavior of these particles allows for the penetration, uptake, and effective delivery of the biomarker to the targeted tissue.

The hyaluronic acid-containing microgels were proposed by Chen et al. to deliver Herceptin, an antitumor protein drug useful for ovarian tumor therapy [197]. The in vitro study demonstrated that the microparticles were hyaluronidase-active while preserving Herceptin conformation and activity. As a consequence, Herceptin was released from microparticles in the SKOV-3 line cells derived from ovarian adenocarcinoma. The cytotoxicity tests showed that hyaluronidase treatment of the cells at a dose of 600 U/mL for 48 h resulted in 100% cell viability. However, the therapy with microgel particles loaded with Herceptin led to cell apoptosis. It was revealed that the hyaluronidase-dependent release of the anticancer drug was almost completed within 10 days, and 80.6% of the drug was liberated from the carrier at 1 U/mL of enzyme. Furthermore, the subcutaneous injection of microgels containing Herceptin cargo led to almost the complete suppression of tumor growth in the mouse model at a dose of 30 mg per equiv./kg. The Herceptin in the microgel formulation turns out to be a much more efficient method to cure the ovarian cancer than the therapy by the drug itself.

### 5.3. Stimuli-Sensitive Carriers for Breast Cancer Treatment

Breast cancer is one of the most common cancers among women. As a result, many strategies have been developed to combat it. Below are examples of stimulus-sensitive drug delivery systems for breast cancer using various types of (biodegradable) polymer, silica, peptide, oligosaccharide, and lipophilic carriers.

A pH-sensitive LbL hyaluronan-containing nanoparticle with a biomarker described in Section 5.2 can also be used for breast tumor treatment [37].

Folic acid-modified PEGylated poly-ε-caprolactone containing a ditelluride linkage and carrying doxorubicin NPs were used to fight against 4T1 breast cancer cells cultivated in vitro [21]. The results of nanoparticle incubation with breast cancer cells revealed that under the influence of local high concentrations of GSH in tumor tissue, the ditelluride bond breaks, releasing the nanoparticles’ cargo into the tumor area. Moreover, it was found that folic acid-decorated nanoparticles facilitated their uptake by target breast cancer cells.

In another study, polylactide-grafted doxorubicin nanoparticles with acid-sensitive labile Schiff-base linkages were used to control drug release in MCF7 breast cancer cells [9]. The study indicates that the NPs were efficiently taken up by breast cancer cells, resulting in effective therapy. The delivery system can be designed to inhibit immunosuppressive lymphocytes, including regulatory T cells and myeloid-derived suppressor cells within the tumor microenvironment. These factors result in defective tumor cells evading surveillance due to the aforementioned immunosuppressive networks within the tumor parenchyma.

Based on these assumptions, Feng et al. elaborated on polypeptide NGs carrying doxorubicin and the immune regulator 1-methyl-D,L-tryptophan to apply chemoimmunotherapy to kill breast cancer cells in vitro in culture and in mice [10]. When the reduction-responsive polypeptide NGs were administered to the tumor, the cargo was rapidly released due to the highly reductive environment in the cancer. The biological studies revealed the effective suppression of tumor growth, with no side effects.

In another study, LPs were used as a support for the LbL deposition of amphiphilic chitosan and hyaluronic acid [12]. The deposition was followed by the loading of survivin-shRNA, an inhibitor of apoptosis, and, subsequently, the permeation promoter hyaluronidase. It is known that silencing the apoptosis inhibitor results in clinically promising and effective breast cancer therapy with low toxicity of the NPs’ cargo [12]. RNAi is a double-stranded RNA interference process in which shRNA (short hairpin RNA) causes gene silencing by targeting and degrading specific mRNA. Moreover, the weak acidic microenvironment of the breast tumor (pH in the range 6.5–6.8) resulted in a change of the particles’ charge (in particular due to the protonation of hyaluronic acid), leading to the release of hyaluronidase and surviving shRNA from the NPs to the extracellular matrix and the tumor cells. As a consequence of gene silencing, tumor cell proliferation was inhibited, as it was demonstrated in cultured cells and in mice. The studies revealed that the concentration of NPs in tumor cells was significantly higher than in normal cells due to stimulus-triggered action. The NPs accumulated and penetrated the tumor, and the cargo was released into the tumor cells.

Another therapeutic method against breast tumors was proposed by Li et al. [126]. The authors developed the hyaluronic acid crosslinked with L-cystine dimethacrylamide NGs containing cytochrome c. Cytochrome c is considered a potent CD44-targeted apoptotic protein. The redox-stimulus system, sensitive to GSH L-cystine –S-S- bond reduction, contained a tri-segmented copolymer composed of hyaluronic acid-grafted–PEG tetrazole and was tested in mice. After intravenous injection, the nanogel particles were internalized by the breast tumor, and the target protein, cytochrome c, was released into the tumor microenvironment under the influence of GSH. The study revealed the efficient inhibition of the tumor growth in mice.

The goal of the anti-cancer therapy cannot be limited to the tumor but also inhibits angiogenesis. The blood vessels that deliver nourishment to fast-proliferating cancer cells are necessary. Yang et al. developed a photothermally sensitive liposome-delivery system composed of NIR light-activated IR780 dye and a hydrophobic Sunitinib drug, which is bound by endothelial tumor receptors and inhibits angiogenesis [163]. Sunitinib is released from the LPs via NIR laser irradiation and targeted to VEGF receptors presented on the endothelial cells. In addition, the IR780 dye emits radiation in the NIR range of the spectrum, resulting in decay of the cancer cells. The IR780–Sunitinib liposomal system was tested on 4T1 breast cancer cells and in a mouse tumor. The study demonstrated that IR780-loaded LPs upon NIR irradiation exhibited cytotoxic effects on 4T1 cancer cells. The viability of the cancer cells decreased to 50% after exposure to LPs containing both Sunitinib and IR780 at a concentration of 0.2 μg/mL and irradiation. The higher IR780 concentration in liposomes resulted in significantly lower viability and greater cytotoxicity. Furthermore, the MVD value, referring to the microvascular density of tumor tissue in mice, was 80.7%, indicating that the simultaneous NIR laser irradiation of IR780 and Sunitinib-loaded LPs in the tumor suppressed angiogenesis. In these conditions, the applied liposomal system was the most effective in fighting the tumor.

The same research group developed a photodynamic liposomal system composed of an IR780 dye and hydrophilic chemotherapeutic tirapazamine located in the lipid bilayer and in the liposome core, respectively [165]. An in vitro study showed that irradiated IR780 dye generated reactive oxygen species and local hypoxia, causing the withdrawal of radicals from tirapazamine and damaging DNA strands in breast tumor cells. In mice, upon photodynamic and chemotherapy, the IR780 and drug-loaded LPs exhibited very potent antitumor activity.

Wu et al. developed an ultrasound-responsive mixture of two commercial triblock copolymers, namely Pluronic P123 and Pluronic P127, composed of poly(ethylene glycol) side blocks and polypropylene oxide central block, in the form of nanomicelles [180]. The nanomicelles served as carriers of hydrophobic curcumin against breast cancer using MDA-MB-231 cells and in 4T1 cancer cells in mice. The study of MCs carrying curcumin demonstrated an encapsulation efficiency of 86.67% for breast tumor cells and prolonged blood circulation compared with free curcumin. After drug-loaded micelle administration into breast tumor cells and focused ultrasound treatment, the local release of curcumin occurred within 5 min. It was found that ultrasound therapy against tumors in mice resulted in a 6.5-fold decrease in the tumor weight compared to tumors treated with Pluronic MCs embedded with curcumin but without sonication.

Liang et al. used cerasomes containing doxorubicin to test the ultrasound-triggered system against adenocarcinoma (MDA-MB-231 cell line) breast cancer in mice [29]. Cerasomes differ from classical LPs in N-[N-(3-triethoxysilyl)propylsuccinamoyl]-dihexadecylamine (CFL), in addition to a pallet of phosphatidylcholine derivatives. They combine properties typical of silica particles and LPs. Thanks to -Si-O-Si- bonds formed between neighboring siloxane chains and stiffening of the surface layer upon sonication. The CFL compound ensures the stability of cerasomes during transport in blood vessels and longer circulation compared to conventional LPs. In addition, it slows down the release of the drug from the cerasome interior to the target cancer cells. In this way, the drug can be released from the cerasomes in a controlled way without a “burst” effect. Under the influence of ultrasound waves, the particles are heated up to 42°C, losing the lipid multilayer, which in turn increases particle permeability and drug release. Thus, release of the drug was controlled by the ratio of CFL to 1,2-dipalmitoyl-sn-glycero-3-phosphocholine (DPPC). The study revealed that a combination of siloxane and lipid chains in cerasome particles favors longer blood circulation. Furthermore, the sonication process results in the controlled slow release of the anticancer drug in the target tissue, ensuring maximum therapeutic efficacy.

### 5.4. Stimuli-Sensitive Carriers for Brain Cancer Treatment

The greatest challenge in treating brain cancer is overcoming the blood–brain barrier (BBB) to deliver the drug. Thus, the drug-carrying vehicle must first cross the BBB, then reach the tumor [245]. In recent years, strategies for delivering drugs across the BBB have been developed, including passive transcytosis, intranasal administration, ligand conjugation with the carrier surface, membrane coating, and BBB disruption using light, focused ultrasound, biochemical reagents, and radiation. The ligands tethered to the carrier’s surface are folate, insulin, transferrin, and low-density lipoprotein and belong to the active targeting strategy. In turn, nowadays, the DDSs for a brain tumor may be composed of red blood cell or tumor cell membranes loaded with an anticancer drug. The mentioned membranes allow the drug-carrying vehicle to circulate in the blood for a long time and to easily penetrate the BBB. In addition, the membranes can be equipped with ligands that enable a target tumor to be achieved more quickly. However, for stimuli-sensitive carriers in DDSs, most often, the amphiphilic copolymers and liposomes are tested.

Cationic poly(β-aminoester) polymer NPs carrying siRNA (small interfering RNA) were used to cure glioblastoma cancer cells in the brain [110]. The siRNA, a short, double-stranded macromolecule, is used in the RNA interference pathway to silence or knock down specific genes and, consequently, protect cancer cells against the encoded protein production. The polymer NPs carrying siRNA were able to knock down specific genes with a small dose of the siRNA equal to 5 nM, compared with a four times higher dose of the commercially available medicinal product Lipofectamine^TM^ 2000.

Thermosensitive LPs containing magnetic Fe_3_O_4_ particles and an anticancer drug—Camptosar—were applied in the therapy of brain cancer (human primary glioblastoma) [129]. In addition, the authors decorated the liposome’s surfaces with cetuximab, an antibody that recognizes epidermal growth factor overexpressed on the surfaces of cancer cells, thereby selectively targeting the cells. It was found that the cetuximab-targeted LPs were taken up by glioblastoma cells cultured in vitro. The following exposition in a high-frequency magnetic field affected the particles’ heating to ca. 43 °C, melting, and release of Camptosar to the cells’ interior. As a result of Camptosar cytotoxic therapy, the cancer cells underwent apoptosis. After cell exposure, cell viability decreased from ca. 80% (at 37 °C) to ca. 40% (at 43 °C) compared with cells exposed to the antitumor drug and Camptosar-free lipid NPs. The effectiveness of a brain cancer therapy was confirmed in a mouse model.

The light-responsive branched pentaerythritol poly(caprolactone)-b-poly(acrylic acid) hydrogel NPs carrying doxorubicin were used to test anticancer activity against C6 glioma brain cancer [171]. The doxorubicin-loaded hydrogel NPs were cross-linked by ferric cations and carboxyl groups derived from poly(acrylic acid). The laser light exposure (at 405 nm) led to decomposition of the NGs, the reduction of Fe^3+^ to Fe^2+^, and the release of ferric cations into the medium containing lactic acid, following the release of doxorubicin. It was found that 82.5% of doxorubicin contained in the NGs was released into the microenvironment within 2 h after drug administration. In addition, the nanogel particles were efficiently internalized by the brain cells cultured in vitro. The cells’ viability was highest (over 80%) for the laser light-treated NGs at the maximum doxorubicin dose. In addition, the drug was released only at the target site without contaminating healthy tissue. In vivo studies using the C6 glioma rat model demonstrated 91.2% inhibition of brain tumor growth in contrast with free doxorubicin administration.

### 5.5. Stimuli-Sensitive Carriers for Liver Cancer Treatment

Liver cancer is difficult to destroy using DDS therapeutics due to the morphology of this organ, strong blood supply, and functions. However, many attempts are being made to propose strategies using single or dual stimulus-sensitive systems for combating liver cancer. Below, one can find a few examples.

Some types of liver tumor cells generate resistance to the anticancer drug, cisplatin. Kang et al. developed an imidazole-modified cisplatin (IV) prodrug also containing a long aliphatic chain (15 carbon atoms) assembled into pH-responsive NPs containing a negatively charged block copolymer composed of polyglutamic acid and PEG [3]. The system was obtained via the electrostatic complexation of modified cisplatin and copolyelectrolyte. The study revealed that pH-sensitive NPs disassembled in the reduced pH of the tumor and overcame hepatocarcinoma (7404 DDP cells) resistance to cisplatin, effectively causing tumor cell death.

The enzyme- and dual-triggered rapid release of an anti-cancer drug from polymer MCs to liver cells was elaborated on by Zhang et al. [115]. The authors proposed camptothecin chemically conjugated with poly(ethylene glycol) via a redox-sensitive disulfide linkage cleavable by GSH. Polyethylene glycol was bound via azo bonds to a poly-ε-caprolactone block. The azo bonds were sensitive to azoreductase and coenzyme NADPH, yielding separated poly-ε-caprolactone and polyethylene glycol segments. The end of poly(ethylene glycol) contained phenylboronic acid, a sensitive compound to enzymes. The results showed that the copolymer MCs were specific to the decomposition of hepatoma carcinoma cells and penetrated cells, liberating camptothecin in the mouse cells’ interior. Furthermore, the polymer MCs manifested high therapeutic efficiency with a survival rate of approximately 100% after 160 days of treatment.

The other group used an approach to kill tumor cells; a static magnetic field was applied to initiate the production of H_2_S bubbles inside tumor cells, blowing them up.

The authors used LPs containing superparamagnetic NPs and a hydrogen-sulfide donor, anethole-trithione, and applied it to kill HepG-2 liver cancer cells cultivated in vitro [140]. The LPs were internalized by liver cancer cells under the static magnetic field. Once the LPs entered cancer cells, enzymatic catalysis resulted in the decay of anethole-trithone and the release of hydrogen sulfide bubbles into the cytoplasm. The H_2_S bubbles increased hydrostatic pressure, leading to unfolding of the cytoskeleton, then bursting and, in turn, the death of the cancer cells.

Functionalized by epithelial growth factor receptor (EGFR), PEGylated LPs carrying chitosan, magnetic particles, and paclitaxel were developed to treat hepatocellular carcinoma liver cancer in mice [141]. The EGFR-functionalized LPs, with efficient drug encapsulation over 90%, under the influence of a magnetic field, reached the target organ and were internalized by cancer cells through binding to the tumor cell surface.

A double stimulus-sensitive system was developed by Cao et al. for HepG2 cancer in mice [162]. Photothermal-responsive PEGylated LPs with a phospholipid bilayer were used to deliver curcumin directly to HepG2 cancer cells upon exposure to NIR laser irradiation. The photothermal drug release, compared with a single thermal stimulus, demonstrated an enhanced therapeutic effect against cancer cells. In addition to curcumin, the LPs were equipped with the photo-sensitive (NIR-absorptive) diketopyrrolopyrrole-based polymer (DPP). The absorption of DPP in the range of wavelength 800–850 nm is advantageous for this photosensitizer in NPs used in vivo because this range is suitable for body irradiation. Since LPs are internalized by tumor cells, exposure to NIR accelerates the release of curcumin upon intracellular decomposition of the NPs. The developed system improved the bioavailability of the hydrophobic drug and promoted controlled release in target cells.

LPs loaded with doxorubicin sensitive to a magnetic field were used to evaluate the drug’s effect on HepG2 liver cancer cells [144]. The study showed the effective entrapment of the LPs by liver cancer cells through the applied magnetic field. The study revealed anticancer activity and targeting efficiency, delivering doxorubicin to liver cancer cells. It was found that doxorubicin-loaded LPs exhibited cytotoxicity, with significant cancer cell viability of approximately 50% and 80% after 24 h and 72 h, respectively.

The complex particle system against HepG2 liver cancer cells, sensitive to the magnetic field, was prepared by Hong et al. [151]. The authors synthesized core-shell NPs containing a magnetite core and a shell composed of polymerized polypyrrole, hyaluronic acid as a linker, and β-cyclodextrins. The doxorubicin hydrochloride was embedded in the nanocomposites’ shells. In the in vitro study, it was demonstrated that doxorubicin was efficiently loaded into the nanocarrier and internalized into HepG2 cells, and enhanced doxorubicin release was achieved under NIR laser light.

### 5.6. Stimuli-Sensitive Carriers for the Colon Cancer Treatment

Colon cancer is one of the most common cancers; however, due to the organ’s accessibility via routes other than the vascular system, there are relatively few reports on stimulus-sensitive DDSs targeting this type of cancer.

It was found that reduction-sensitive polypeptide nanogels carrying doxorubicin and the immune regulator 1-methyl-D, L-tryptophan, as described in Section 3.3, effectively destroy colon cancer cells cultured in vitro and in mice [10].

In turn, low-frequency ultrasound-triggered LPs embedded with cisplatin were used to cure C26 adenocarcinoma colon tumor in mice [185]. The study demonstrated that after 24 h from the injection of LPs containing cisplatin cargo and sonication of the target tumor site, the therapeutic effect was compared with that achieved in the control group of mice not treated with ultrasound but treated with LPs. In the case of the short-time ultrasonication of localized LPs loaded with cisplatin, the colon tumor cells stopped proliferating, and the tumor started to disappear. This tumor regression indicated that local cisplatin release at the tumor site was devoid of side effects in other organs.

### 5.7. Stimuli-Sensitive Carriers for the Bone Cancer Treatment

Bone cancer is one of the tumors that should be diagnosed early. In addition, access to the tumor localized in bone is difficult due to the bone morphology. Despite these challenges, strategies for delivering stimulus-sensitive drugs to the bone are being developed. Examples of the selected drug carriers using a stimulus-sensitive strategy are described below.

Redox-sensitive cationic LPs decorated with hyaluronic acid and cholesterol conjugated via a disulfide linker, equipped with poly(ethylene glycol) at the surface, were used to fight against osteosarcoma in vivo [121]. The disulfide linker was prone to cleavage in glutathione, which is present at an abundant concentration in cancer tissue. The hyaluronic acid acted as a ligand to a large number of CD44 receptors expressed on the surfaces of cancer cells. Redox-sensitive LPs loaded with doxorubicin were used to evaluate anticancer activity against osteosarcoma. The burst release of 60% of doxorubicin was determined in cells in the presence of 10 mM glutathione. In addition, hyaluronic acid-coated LPs showed a 10-fold increase in the half-life of doxorubicin in the cancer microenvironment compared with free doxorubicin in a study in rats. The entire population of mice and rats survived after redox-sensitive cationic LPs carrying doxorubicin, in contrast to those with carrier-free doxorubicin.

Another group of researchers developed a dual-targeting, redox-sensitive liposomal system containing doxorubicin and the tumor-penetrating tripeptide (iRGD) for the treatment of osteosarcoma [10]. Hyaluronic acid was a ligand for cluster of differentiation 44 (CD44), whereas alendronate was a bone target. The iRGD peptide enabled more efficient tumor penetration. The doxorubicin delivery system was composed of an alendronate and hyaluronic acid conjugate, altogether bound to a lipid conjugated with PEG and containing a disulfide linker cleavable by glutathione. It was found that co-administration of tumor-penetrating peptide and bone-targeting factors resulted in effective therapy and the suppression of tumor growth in mice.

### 5.8. Stimuli-Sensitive Carriers for Pulmonary Cancer Treatment

The occurrence of pulmonary melanoma in humans is extremely rare. Presumably for this reason, relatively few reports on the development of stimulus-sensitive DDS strategies to combat this cancer are described. We present the selected example.

The liposomal reduction-sensitive system containing doxorubicin as a therapeutic agent for pulmonary melanoma was prepared by Tian et al. [123]. The LPs were functionalized with heparin (LMWH) to prevent tumor metastasis, increase uptake by tumor cells, and protect from migrating to other organs. The mentioned comprised heparin (LMWH) covalently bound to doxorubicin via a cleavable –S-S- linkage, and the drug, in turn, was covalently bound to phosphatidylcholine in LPs. The anticancer activity of the LPs was evaluated in the murine melanoma cell line and mice. The study showed a burst release of doxorubicin from the LPs in pulmonary tumors and lower side effects in heparin-containing LPs compared to doxorubicin alone in LPs.

### 5.9. Stimuli-Sensitive Carriers for Cervical Cancer Cells (HeLa Cells)

Cervical cancer is one of the most investigated cancers in women. Nowadays, they are used in various laboratory tests to serve as reference tumor cells. Thus, numerous DDSs were elaborated on using HeLa cells, including stimulus-sensitive DDSs involving phospholipids, oligosaccharides, silica, and synthetic copolymers. Here, we cite just a few examples.

Magnetic field-sensitive PEGylated LPs with embedded magnetic NPs loaded with doxorubicin were used in vitro to test this material as an anticancer therapeutic agent in HeLa cells [22]. The HeLa cell line, derived from cervical cancer found in Henrietta Lacks in 1951, was taken out and thoroughly reinvestigated. Due to their extensive proliferation and handling, the HeLa cell line has been kept alive and is immortal. Thus, it is known that doxorubicin kills the HeLa cells. The study revealed that when exposed to a high-frequency magnetic field, PEGylated LPs melted and released doxorubicin, killing HeLa cells. The system was not harmful to (healthy) fibroblast L-929 cells. Moreover, the cytotoxicity of doxorubicin was proportional to the drug concentration in the LPs.

Similar to the study on the anti-cancer activity of magnetic PEGylated liposomes with doxorubicin, Pradhan et al. tested the therapeutic action of folate-decorated magnetic PEGylated LPs with doxorubicin cargo on HeLa cells [134]. The hyperthermia triggered the release of the drug, affecting doxorubicin release at 43 °C. At this temperature, 52% of loaded doxorubicin was released from the LPs in 50% fetal bovine serum after 1 h of incubation.

The light-sensitive amphiphilic MCs composed of hyaluronan-o-nitrobenzyl-stearyl copolymer chains loaded with doxorubicin were tested to inhibit the proliferation of the CD44-overexpressing HeLa cells [156]. The study demonstrated that the target cells effectively internalized the MCs due to the presence of hyaluronan receptors on their surface. Irradiation with UV light (365 nm) disassembled the MCs, leading to drug release at the target site.

It is well known that active targeting and selectivity promote the highly localized binding and uptake of anti-tumor nano- and microparticles and improve the effectiveness of the therapy.

Cheng et al. elaborated on complex mesoporous silica microparticles loaded with doxorubicin in their mesopores for controlled, targeted anticancer therapy against cervical cancer [201]. To target tumor cells, the microparticles’ surface was equipped with a rotaxane tether α-cyclodextrins, followed by covalent binding of the tri-segmented peptide GFLGR_7_RGDS (Gly-Phe-Leu-Gly-Arg_7_-Arg-Gly-Asp-Ser). The tri-peptide combined tumor-targeting, membrane-penetrating, and cathepsin B-responsive functionalities. Cathepsin B is a cysteine protease acting in lysosomes and is overexpressed in various tumor cells. A tumor-targeting RGDS ligand recognizes the α_v_β_3_-integrin receptor on tumor cells, and the R_7_ sequence enables tumor cell penetration. In turn, the cathepsin B-cleavable GFLG segment enables the release of doxorubicin into the cytoplasm, leading to tumor cell death. An in vitro study showed that 60% of encapsulated doxorubicin was released at physiological conditions, pH = 7.4, in the presence of cathepsin B within 24 h. The cytotoxicity tests in HeLa cells revealed very good biocompatibility of the polysiloxane particles free of doxorubicin and good toxicity of the same functionalized particles loaded with doxorubicin. The IC_50_ values for doxorubicin and doxorubicin embedded in the functionalized silica microparticles in HeLa cells were equal to 1.2 and 4.3 μg/mL, respectively. In turn, the viability of the HeLa cells after the administration of a free anticancer drug and the drug in the functionalized silica carrier was similar, ca. 20%. However, due to the selectivity of doxorubicin embedded in silica microparticles, the encapsulated drug did not cause toxic effects to normal cells.

### 5.10. Stimuli-Sensitive Carriers for Pancreatic Cancer

Pancreatic cancer belongs to poorly permeable and drug-resistant cancers, and thus, the drug therapy is highly impaired. Li et al. reported a stimuli-responsive poly(amidoamine)-graft-poly-ε-caprolactone containing free poly-ε-caprolactone and poly(ethylene glycol-b-poly-ε-caprolactone) complex clustered system that overcomes drug delivery barriers to that organ [39]. The authors synthesized raspberry-like NPs that circulate in the blood and accumulate in the pancreatic tumor. The acidic microenvironment of the tumor triggers the structural modification of the nanoparticle via the discharge of platinum prodrug-conjugated polyamidoamine DDRs. The DDRs comprise covalently bound cisplatin, leading to the release of 5 nm nanoclusters containing the prodrug. The nanoclusters were able to penetrate pancreatic tumor cells, once internalized, and release cisplatin at the acidic pH of the cytosol, killing tumor cells. The in vitro study visualized differences in nanocluster uptake by pancreatic tumor cells, leading to apoptosis. It was found that after 4 h of nanocluster incubation with tumor cells, the raspberry-like NPs were partially deformed, and after 24 h, they completely disintegrated into 5 nm NPs. The study of tumor apoptosis resulting from platinum prodrug raspberry-like NP treatment showed that 36.8% of cells were killed. However, nearly 14.3% of tumor cells were killed when treated with NPs embedded with physically bound cisplatin. In a mouse model, the nanocluster antitumor activity showed 88% suppression of tumor growth compared to cisplatin-free nanoclusters.

Dwivedi et al. proposed complex magneto-liposome microbubbles containing doxorubicin and equipped with magnetic particles to kill pancreatic tumor cells [147]. The authors prepared citrate-stabilized iron oxide NPs and doxorubicin, both internalized in liposomes. The cargo-loaded LPs were covalently conjugated to gas-loaded hydrophobic perfluorocarbon microbubbles. The whole complex system, upon exposure to pulsed ultrasound, showed very good transport to cancer cells and the release of doxorubicin, leading to apoptosis of the tumor cultivated in vitro. Moreover, a significant reduction in tumor volume was observed in the group of mice with pancreatic cancer treated with microbubbles compared with the group of mice deprived of therapy.

In another study, a modified nanoemulsion containing perfluorocarbon was used to combat pancreatic cancer in cell culture (using MiaPaCa-2 cells) and in mice [179]. The emulsion nanodroplets were prone to ultrasound and acted as fluorine markers when monitored via ultrasonography. The authors used perfluoro-15-crown-5-ether to prepare a nanoemulsion due to the linear equivalent instability. In turn, perfluoro-15-crown-5-ether was localized to the particle interior, loaded with paclitaxel, and stabilized with one of three tested block copolymers, such as PEG-b-poly(D,L-lactide), PEG-b-poly(L,L-lactide), or PEG-b-poly(ε-caprolactone). The therapeutic sonication with 1 MHz yielded from nanoemulsion nanobubble–perfluorocarbon-copolymer droplets due to vaporization of the latter under heat. The study revealed that stable nanobubbles carrying paclitaxel were circulating in the blood and accumulated in the pancreatic tumor.

The sensitive enzyme-stimulus system against pancreatic cancer was prepared by Shchegravina et al. [188]. The system consisted of LPs containing colchicinoids in their bilayer, which are considered promising prodrugs for pancreatic tumor therapy. The liposomal system was designed to fit the phospholipase A2-binding site, which is abundantly present in cancer tissue. The colchicinoid prodrug was covalently bound to the lipid chain via an ester linkage prone to nonspecific esterase hydrolysis, such as phospholipase A2. The enzyme can hydrolyze lipids specifically at the sn-2 position, leading to the LPs’ decay and the release of the drug, in this case, colchicine. In the next step, hydrolysis of the drug–lipid bond leads to the liberation of colchicine in the tumor cells’ interior. The results of cytotoxicity tests on pancreatic cancer cells treated with drug-loaded LPs showed high cytotoxicity of the colchicin derivative–lipid conjugates, and the IC50 after 72 h of incubation of the LPs with the cells was 38 nM.

The matrix metalloproteinase-2-sensitive (MMP-2) combined liposomal system was developed by Ji et al. [189]. Due to the difficulty of efficient drug penetration into pancreatic cancer tissue, the system comprised an antifibrosis drug and an antitumor drug in one nanoparticle. Moreover, it exhibited disassembly in the tumor tissue into two segments. The one segment comprised β-cyclodextrin attached to pirfenidone, the antifibrotic drug, decreasing the barrier of the stroma. The surface of the other one contained an RGD (Arg-Gly-Asp) tripeptide, the anticancer drug gemcitabine. The tripeptide was specifically bound to the tumor cell surface. Once the LPs entered the tumor, the matrix metalloproteinase-2, presented in the tumor tissue, cleaved the β-cyclodextrin-pirfenidone linkage. Then, pirfenidone interacted with the pancreatic stellate cells, facilitating the access of liberated gemcitabine to the tumor cells. The elaborated liposome system was tested on pancreatic tumor cells and on the xenograft mouse model of pancreatic tumor. The cytotoxicity test revealed that the LPs loaded with gemcitabine and equipped with RGD tripeptide, prone to cleavage, exhibited the greatest cytotoxicity among the analyzed combinations, compared to free gemcitabine. The lowest tumor cell viability, approximately 10%, was observed for LPs containing the RGD and β-cyclodextrin–pirfenidone complex loaded with 10 μM gemcitabine and incubated with cells for 24 h. The two-week treatment of mice with the liposome system resulted in a significant decrease in the tumor size compared to mice treated with other formulations, including free gemcitabine.

### 5.11. Stimuli-Sensitive Carriers for Prostate Cancer

Prostate cancer is a common malignancy of the prostate gland that affects men. There are a few classical methods for fighting; however, there is a growing search for safer and less invasive strategies, including stimulus-sensitive DDSs. The selected examples are listed below.

Hybrid thermoresponsive NGs composed of poly(ethylene glycol) methyl ether methyl methacrylate and poly(methacrylic acid), loaded with magnetic NPs and maghemite, were used to induce local magnetic hyperthermia to control the remote release of doxorubicin in prostate cancer cells [138]. Although the material is presented under the name “nanogels” (NGs), according to the IUPAC nomenclature of particles, it is referred to as a microgel particle [246]. Under physiological conditions, the NGs exhibit swelling–deswelling behavior at 47 °C, far from the normal body temperature (36.6 °C). However, exposing human prostate cancer PC-3 cells to NGs loaded with doxorubicin in an alternating magnetic field caused the NGs to shrink. It led to a 2-fold increase in drug release, without overall heating of the cells. Under these conditions, the increased intracellular release of doxorubicin from the NGs enhanced cytotoxicity in cancer cells, resulting in 47% of cells undergoing apoptosis. It is worth mentioning that barely 26% of the cells underwent apoptosis when the alternating magnetic field was not applied.

In turn, the complex particles were prepared as gold nanorods coated with trimethylcetyl ammonium bromide (CTAB), which carries a positive charge and helps ensure stability [153]. The CTAB multilayers were deposited on the gold nanorod surface during nanoparticle manufacture. The CTAB served as a gold nanoseed capping from which the nanorods were obtained in seed-mediated growth [220]. The NPs served as an imaging tool to monitor tumor growth. The developed NPs exhibited the Arrhenius-like photothermal response during the hyperthermic ablation of prostate (PC3-PSMA) cells [154]. Due to poor stability in the biological environment and the low loading capacity of DNA in CTAB-gold nanorods, the authors used multilayer polyelectrolyte-labelled gold nanorods. The polyelectrolyte shell was composed of EGDE-3,3′-PSS-CTAB layers, in which the most outer layer—EGDE-3,3′ denoted the product of the ring-opening polymerization of ethylene glycol diglycidyl ether and 3,3-diamino-N-methyldipropylamine, PSS—poly(styrene sulfonate). According to the authors, the polyelectrolyte-encapsulated gold nanorods overcome these problems; therefore, the DNA-loaded gold nanorods were effectively used for DNA transfection in cancer cells.

### 5.12. From the Nano- and Microparticle Formulations of Anticancer Drugs to Preclinical Studies

In general, researchers developing DDSs (not limited to stimuli-responsive carriers) highlight the following point. Whereas the vast majority of publications in this field present basic study results, only very few have reached the clinical study level, and only some of them have entered the market. It seems to us that the explanation of such a situation depends not so much on medicine, pharmacy, and chemistry, but on economics. The research on carrier synthesis and characterization is relatively inexpensive. The research involving cell cultures costs much more. However, the significant rise in development cost begins with unavoidable studies that require animal models (most often mice or rats). The last stage is to obtain approval of a regulatory agency (such as the US Food and Drug Administration (FDA) or European Medicines Agency (EMA)); however, there are also regulatory agencies at the country level. To put a drug on the market requires at least phase I and phase II clinical studies. The whole process may take longer (sometimes years) and consume enormous financial resources, which only large pharmaceutical companies can afford. However, in some instances, the time for bringing a product to market has been very short. This is the case with the COVID-19 vaccine, which was available within a few months of the pandemic’s start. In the case of anticancer DDSs, progress is slow and moves step-by-step. However, it is worth noting that the development of some cancers (e.g., cervical, vaginal, penile, and anus) can be induced by oncogenic types of human papillomavirus (HPV). Fortunately, vaccination against HPV eliminates the risk of the aforementioned cancers.

The bioactive substances used to prepare the anticancer drugs are usually toxic not only to cancer cells but also to healthy cells. Therefore, to reduce the undesired and often severe side effects, the drugs should be precisely delivered where needed, and the bioactive substances should be released on demand. Section 5 describes many examples of such materials and their properties. If the stimuli-sensitive materials do not interact with a given bioactive substance, the approval of the new stimuli-sensitive drug formulation should be easier, requiring only approval of the auxiliary material.

Translating research findings from the animal model phase (e.g., studies in rats or mice) into clinical trials is critical. The reason is very simple. Obviously, humans are not rats. Therefore, even if the results of animal studies are very positive, researchers would know how to treat rats, but not humans. By the start of clinical trials, all results from animal studies are already known. However, the therapeutic dose cannot be adjusted based on a simple body weight ratio; rather, it must be determined according to the protocol used in clinical trials. Furthermore, the immune system response in animals and humans may differ. In addition, the characteristics of animal models are highly standardized, whereas the human population is much more heterogeneous. Another issue is the delayed onset of the drug’s effect, which must be monitored, even though this requires more time. For ethical reasons, pregnant women are excluded from clinical trials, as chemotherapy can be harmful to the fetus.

### 5.13. The Preclinical Studies and Clinical Trials of Nano- and Microparticle Formulations of Anticancer Drugs

Despite many years of research on the target systems for controlled drug delivery, few formulations have been introduced to the market. There are various reasons for such a situation. The new formulations must fulfill very strict requirements to be accepted for production on a larger scale, and each step of their manufacturing and storage should be well-controlled. The effectiveness of the target DDS must repeatedly exceed that of the carrier-free anticancer drug. It should also be noted that each new polymer should be tested for immunogenicity and inflammation. Polymers that do not exhibit such properties may induce immunogenicity after the use period (e.g., as happened with PEG).

As it was mentioned, testing the new drug requires significant financial investment (typically ca. 1 mld. Euros) in preliminary studies that typically last many years (ca. 8–12) and involve a large number of samples. In preclinical studies, the new drug is tested in at least two animal species (e.g., rats and dogs), and it takes ca. 5–8 years, in this stage, how the drug interacts with a living organism and whether it is harmful. The studies at this stage aim to confirm the drug’s effects, identify adverse effects, and investigate its absorption, metabolism, and excretion. Then, four clinical trials are conducted on numerous groups of volunteers [247]. In each step of the studies, some drugs are eliminated due to various side effects. The goal of the first phase is to determine the safety and dosing of the new drug. They involve ca. 20–100 healthy and sick volunteers. In the next (II) stage, several hundred people participate, and the effectiveness and adverse effects of the potential drugs are being checked. In the third (III) phase, the drug is tested on a significantly larger group of suffering volunteers in the range of 300–3000. Using this group, the effectiveness and side effects are further verified. In phase IV, 25–30% of drugs that passed the previous phases are further tested in a few thousand people with the target disease, and their effectiveness and safety are once again verified. Finally, because the selected drug complies with the entire specified protocol, it undergoes a longer registration process, during which numerous analytical analyses and reports on effectiveness, safety, and drug quality are required.

In the literature, reports on anti-cancer drug formulations, such as polymeric, liposomal, dendritic, or conjugated to a polymer chain, that have passed clinical trials and been accepted by the FDA are not very numerous compared to the number of papers published on this topic in the last few decades. These studies on novel formulations are in demand as the number of people suffering from various cancers increases.

In Table 3, exemplary stimulus-sensitive carriers of anticancer drugs are listed, along with the corresponding preclinical or clinical study stages. It is worth noticing that carriers involved in the described delivery systems belong to various chemical groups, such as natural, natural but chemically modified [248], and synthetic polymers, which belong to proteins [249], oligosaccharides [248,250,251,252,253,254], biodegradable PEGylated polyesters [247,255], lipids [256], hydrophilic polyethers (PEG) forming conjugates with drug [257,258], and polyamides [247,259].

## 6. Conclusions

An overview of the stimulus-sensitive polymer DDSs is presented. The reference list is not exhaustive but focuses on the main types of stimuli used to prepare drug carriers, including the temperature, pH, electromagnetic fields, ultrasound, and enzymes. The detailed chemical structures of the anticancer drug carriers are presented, along with the basic principles of their operation after administration to the body. The most commonly applied stimulus is pH; other stimulus-responsive systems are reported less frequently. However, there are unresolved issues that require finding solutions in the near future.

Cancer affects the entire body due to immune system dysfunction or the penetration of certain viruses into tissues. In many cases, identification of the cause is very difficult.

Furthermore, a serious disadvantage of the tested anticancer systems is the lack of the transferability of therapeutic effects from animal models to human tumors. The morphology of tumors generated in animals is completely different from that of human tumors. The tumors generated in animals are much more solid than those in humans, which are more able to penetrate through blood vessels. Therefore, delivery systems with slightly different properties are needed.

Along with the development of stimulus-sensitive carriers, an increase in novel stimulus-responsive multi-functional devices will be evaluated. Together with the new drug carriers, the number of functional polymers, the number of cleavable chemical bonds, and the number of stimuli should increase. The same applies to hydrophilic coatings of MCs, NPs, or LPs, which are currently mostly composed of PEG. Due to the widespread use and increased number of adverse effects, PEG substitutes should be elaborated and established as therapeutic agents. However, the long-term testing of new DDSs across various aspects is conducted due to a limited understanding of how these materials affect the human body.

The fundamental issue in drug delivery systems, aside from non-toxicity, is the transport to the target tissue while sparing healthy tissues from the toxic action of an anticancer drug. Thus, it is advisable to decrease the ratio of toxicity to cancer to toxicity to healthy tissues in the body.

In addition, drug delivery systems are developed to destroy primary tumors; however, metastatic (secondary) tumors cause many more problems, so that early detection and removal should be intensified.

It must be stressed that, despite numerous efforts and years of research on various drug delivery systems, including modern single- or double-stimulus-sensitive systems for cancer, this problem remains unsolved and is far from a complete solution.

## Figures and Tables

**Table 1:** Factors of the stimuli-responsiveness: pH, temperature, redox potential, electromagnetic field, ultrasound, and catalytic enzymes, and corresponding references.

Factor/Stimulus	Factor Site	References
pH	Internal	[3,9,30,31,32,33,34,35,36,37,38,39,40,41,42,43,44,45,46,47,48,49,50,51,52,53,54,55,56,57,58,59,60,61,62,63,64,65,66,67,68,69,70,71,72,73,74,75,76,77,78,79,80,81,82,83,84,85,86,87,88,89,90,91,92,93,94]
Temperature	Internal	[15,31,94,95,96,97,98,99,100,101,102,103,104,105,106,107,108,109,110,111,112]
Redox potential	Internal	[4,10,11,12,13,19,21,91,113,114,115,116,117,118,119,120,121,122,123,124,125,126]
Electromagnetic field	External	[22,127,128,129,130,131,132,133,134,135,136,137,138,139,140,141,142,143,144,145,146,147,148,149,150]
Light	External	[91,151,152,153,154,155,156,157,158,159,160,161,162,163,164,165,166,167,168,169,170,171]
Ultrasound	External	[24,29,172,173,174,175,176,177,178,179,180,181,182,183,184,185,186]
Enzymes-catalytic	Internal	[2,50,115,187,188,189,190,191,192,193,194,195,196,197,198,199,200,201,202,203,204,205,206,207,208,209,210,211,212,213,214]

**Table 2:** Overview of the morphology of drug carriers.

Carrier	Type of Structure	Characteristic Features	References
Macromolecular carrier	Dendrimers (DDRs)	Size below 100 nm. DDRs are single organic macromolecules with a hierarchical architecture, usually containing PEG on the surface. DDRs may also form crosslinked multiaggregates that can disintegrate quickly in response to a stimulus.	[5,6,7,214]
	Nanogels (NGs)	Size below ca. 200 nm. NGs are prepared from crosslinked synthetic polymers, oligopeptides, and oligosaccharides and may be coated with PEG and/or ligand-binding receptor on tumor cells. NGs may be multi-stimuli responsive. They disintegrate in a controlled manner in response to a stimulus, triggering drug release.	[16,86,87,88,89,90,112,124,126,138,148,149,150,171,214]
Micelles (MCs)		Size below 100 nm. Type of nanoparticles with good biodegradability. MCs are prepared from amphiphilic di- or multiblock copolymers, often with PEG or oligosaccharides as a hydrophilic block. They may contain a targeting ligand at the surface and can be loaded with magnetic particles; they disintegrate in a controlled manner in response to thermo- or electromagnetic stimuli.	[54,55,56,68,80,81,82,83,95,115,136,156,179,180,181,184,211]
Nanoparticles (NPs)		Size below 100 nm. NPs may be biocompatible and biodegradable. NPs are prepared from synthetic polymers (polyesthers, polyethers), natural polymers (oligosaccharides, oligopeptides), or di- or multiblock copolymers, often crosslinked, and may contain PEG and/or ligand-binding receptor on tumor cells. They may contain embedded superparamagnetic NPs or bounds, susceptible to hydrolysis, pH changes, or irradiation. They disintegrate in a controlled manner.	[2,3,4,9,38,42,48,49,50,53,57,58,59,68,76,79,92,99,114,125,127,132,133,135,136,137,146,160,169,170,186,191,192,201,206,208]
Multilayer (LbL) capsules (MCPs)		Size below 500 nm. MCPs are composed of a metal (gold) particle(s) core and polyelectrolyte shell. The shell is prepared using a layer-by-layer (LbL) technique from two polyelectrolytes containing opposite charges. MCPs offer the possibility of embedding drugs between multilayers. They disintegrate in a controlled manner in response to hyperthermia.	[37,51,95,151,152,153,154,226,227,228,229,230,231,232,233,234,235,236,237,238,239,240,241,242]
Liposomes (LPs)		Size in the range 0.01–1 μm. LPs are biocompatible and composed of phospholipids forming bilayer vesicles filled with liquid. They often contain cholesterol to protect the anticancer drug against leakage from particles. LPs may contain magnetic NPs and PEG or ligand-binding receptors on tumor cells. They are susceptible to quick melting in response to hyperthermia. LPs are most commonly used in the treatment of cancer in developed pharmaceuticals.	[11,14,30,31,32,33,63,64,65,66,69,70,71,72,74,75,97,98,101,102,103,104,105,106,107,108,109,110,121,122,123,129,133,140,141,142,143,144,145,147,155,156,162,163,164,165,166,167,176,177,178,183,185,188,189,190,191,198,199,200,207,210,243,244]

**Table 3:** Exemplary tumor-targeted stimuli-sensitive carriers at various stages of studies in preclinical and clinical phases.

Organ, Type of Cancer	Carrier	Stage of the Studies	Ref.
Breast cancer	Albumin formulation (Abraxane^®^ ABI-007) with Paclitaxel	III Phase	[249]
Breast cancer	Carboxydextran-coated superparamagnetic iron oxide (MagSnow)	II Phase	[248]
Breast cancer	1% glycated chitosan NPs	III Phase	NCT03202446 [250]
Breast cancer	Chitosan hydrogel	Preclinical	[251]
Breast cancer	Genexol-PM (PEG_2000_-b-PDLLA_1750_ 25 nm polymer MCs)	III Phase (completed)	[247]
Metastatic breast cancer	Genoxol-PM (PEG_2000_-b-PDLLA_1750_) with Doxorubicin	II Phase (unknown)	[247]
Breast cancer	NK105 85 nm MCs (PEG_12000_-b-poly (4-phenyl-1-butanoate-L-aspartamide)_8000_) vs. Paclitaxel	III Phase (completed)	[247]
Breast cancer (advanced)	Nanoxel-PM (PEG_2000_-b-PLA_1765_, 25 nm MCs) with Mitomycin-C	I Phase (unknown)	[247]
Metastatic cancer	Nanoxel-PM (PEG_2000_-b-PLA_1765_, 25 nm micelles) with Mitomycin-C	III Phase (terminated)	[247]
Ovarian cancer	Genoxol-PM (PEG_2000_-b-PDLLA_1750_) with Carboplatin	I Phase	[247]
Bladder cancer	Genoxol-PM(PEG_2000_-b-PDLLA_1750_)	II Phase (completed)	[247]
Hepatocellular carcinoma cancer	Genoxol-PM(PEG_2000_-b-PDLLA_1750_)	II Phase	[247]
Non-small cell lung cancer	Genoxol-PM (PEG_2000_-b-PDLLA_1750_) with gemcitabine	II Phase (completed)	[247]
Gastric cancer	NK105 85 nm MCs (PEG_12000_-b-poly(4-phenyl-1-butanoate-L-aspartamide)_8000_) with Paclitaxel administered every 3 weeks	II Phase	[247]
Liver cancer	Alginate-SS-Ibuprofen derivative MCs	Preclinical	[252]
Brain glioblastoma multiforme cancer	Pullulan crosslinked with poly(deca-4,6-diynedioic acid) NGs loaded with temozolomide and indocyanine green	Preclinical	[253]
Cancer	Acetalated dextran-protein (HRP) ** bioconjugate with indole-3-acetic acid prodrug	Preclinical (in 2023)	[254]
Breast cancer	PLGA in situ gel	III Phase	[255]
Prostate cancer	PLGA microparticles	III Phase	[255]
Colorectal cancer	(Nano)liposomes with irinotecan and its active metabolite SN-38 obtained by the activity of carboxylesterase	Preclinical (in 2014)	[256]
Gastric and gastroesophageal cancer	PEG-camptothecin conjugate with PEG 40 kDa through C-20 hydroxyl using glycine as a spacer	Entered clinical studies. It was discontinued in phase II due to low efficacy and toxicity issues.	[257]
Breast cancer	Multiarm (4-arms with 40 kDa) conjugate PEG-SN38 using glycine as a spacer with camptothecin	Under a multiple-phase II trial	[257]
Ovarian, breast, colorectal, and cervical cancer	PEG-irinotecan conjugate containing cetuximab	In phase I, 1.2–6.5 folds. Under the phase II trial, higher cumulative exposure to irinotecan	[258]
Various tumors	Poly N-(2-hydroxypropyl)-methacrylamide (polyHPMA)-based conjugate with dia- minocyclohexane platinum (Pt) bound via pH-sensitive linker	In the phase II trial	[259]
Lung carcinoma, a solid tumor	NK105 * (polymeric micellar NPs) with Paclitaxel combined with radiation	Clinical trial (in 2006)	[260]

* NK105 NPs composed of amphiphilic diblock copolymer in which PEG was a hydrophilic block, polyaspartate block with carboxylic groups modified with 4-phenyl-1-butanol, and 50% of the groups were converted to 4-phenyl-1-butanolate. ** Acetalated dextran–protein (horse radish peroxidase, HRP) conjugate—HRP activates the indole-3-acetic acid by oxidation into radicals, leading to cellular apoptosis.

## Data Availability

No new data were created or analyzed in this study. Data sharing is not applicable to this article.
